# Probing dynamic oxygen exchange for hydrogen production with operando neutron diffraction

**DOI:** 10.1038/s44286-025-00231-9

**Published:** 2025-06-04

**Authors:** Daniel M. Telford, Alex Martínez Martín, Matthew D. Guy, Paul F. Henry, Martin O. Jones, Wenting Hu, Ian S. Metcalfe, John S. O. Evans

**Affiliations:** 1 School of Engineering, Merz Court, Newcastle University, Newcastle upon Tyne, UK; 2 ISIS Neutron and Muon Source, Science and Technology Facilities Council, Harwell Campus, Rutherford Appleton Laboratory, Didcot, UK; 3 Department of Chemistry, Durham University, Lower Mount Joy, Durham, UK

**Keywords:** Engineering, Chemical engineering, Chemical engineering, Materials for energy and catalysis, Characterization and analytical techniques

## Abstract

A chemical looping process exploiting the variable oxygen content of ABO_3−*δ*_ perovskite materials can achieve super-equilibrium conversions of societally important reactions such as the water–gas shift reaction (CO + H_2_O ⇋ CO_2_ + H_2_). The approach relies on an evolving oxygen chemical potential gradient within a reactor bed. Here we show that the oxygen-sensitivity of operando neutron powder diffraction experiments can reveal how the reactor functions with high spatial- (≲1 cm) and time- (≲30 s) resolution. We show how this operando method enables rapid testing of new high-capacity bed materials without previous knowledge of their thermodynamic properties, and gives direct information on their long-term stability. We introduce how this memory reactor concept can also be applied to the steam methane reforming reaction (CH_4_ + H_2_O ⇋ CO + 3H_2_), the key preprocess to the water–gas shift reaction in H_2_ production.

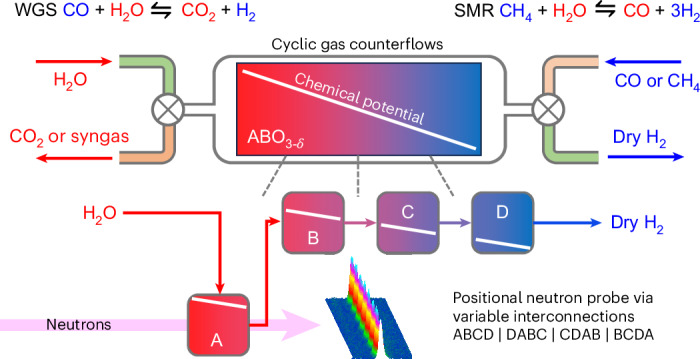

## Abstract (web summary)

Efficient hydrogen production is a major societal challenge. Here the authors use operando neutron diffraction to quantitatively support the operating principle of a memory reactor that allows super-equilibrium operation of the water–gas shift reaction, which can also be used for steam methane reforming.

## Main

Improving the efficiency and sustainability of key chemical transformations remains a major societal challenge, and will be crucial in achieving net-zero targets. The water–gas shift reaction (WGS) (CO + H_2_O ⇋ CO_2_ + H_2_) is one such transformation. WGS is a critical step in hydrogen production, where it is performed globally on a multi-million ton per year scale^1^. It is often operated as a follow-up process to steam methane reforming (SMR) (CH_4_ + H_2_O ⇋ CO + 3H_2_) to increase H_2_ yield. As WGS is a reversible reaction, it is particularly important to be able to drive it to conversions greater than the equilibrium value^2,3^ (super-equilibrium operation) to reduce cost, separation duties and the need for recycle. We detail here studies on the recently described ‘memory reactor’^4^ or ‘regenerative reactor’^5^ concept that exploits the variable oxygen content of ABO_3−*δ*_ perovskite oxygen-carrier materials (OCMs), and the gradient in oxygen chemical potentials that can be developed in them, to achieve this. Conversions can be driven arbitrarily close to 100% and separate product streams are obtained, allowing the development of very different hydrogen production processes. We report how operando neutron powder diffraction studies can give precise atomic-level insight into the reactor operation. Finally, we introduce how the memory reactor concept can be developed for SMR and describe the evolution of such a system over hundreds of operating cycles.

Neutron diffraction was selected for the WGS study because of its high sensitivity to the oxygen atom content of the OCM and its ability to penetrate bulk samples housed within industrially relevant reactor vessels^6^. We describe how the method provides direct evidence of the operating principle on which the memory reactor relies by showing the presence of an oxygen chemical potential gradient in a working reactor. This is achieved without the need for a priori knowledge of the thermodynamic properties of the OCM. We also show how real-time atomic-level information can be obtained on a working reactor bed at a time resolution <30 s, allowing the direct observation of changing bed composition during operation. All this has been achieved via a design concept for neutron studies, which effectively allows a high-intensity white neutron beam to be scanned along the length of a working reactor bed giving both spatial and temporal resolution.

**Fig. 1:**
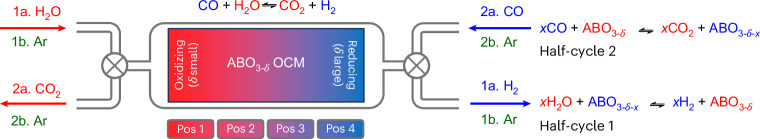
The memory reactor. The memory reactor concept breaks the overall WGS down into two half cycles. In half-cycle 1, H_2_O is used to oxidize a nonstoichiometric perovskite OCM and is reduced to H_2_ (flow 1a). If needed, these gases are then flushed out with Ar (flow 1b). In half-cycle 2, CO reduces the OCM and is itself oxidized to CO_2_ (flow 2a) then flushed (flow 2b). Product separation is achieved by valve switching. By limiting the OCM conversion in each half-cycle, a smooth gradient in oxygen content develops in the OCM and overall gas conversions far in excess of those under thermodynamic conditions can be achieved. In the LSF CL experiments described, the bed consisted of around 100 g of ~1-mm spherical beads. In this and other figures, oxygen-richer species are drawn in red and oxygen-poorer in blue. Boxes labeled Pos 1 to 4 introduce the split-bed concept described in Fig. 2. Pos, position.

Our memory reactor concept is illustrated in Fig. 1 and relies on a chemical looping (CL)^7^ process with a bed of a nonstoichiometric ABO_3−*δ*_ perovskite as an OCM^8–11^. When operated under counter flow, with H_2_O and CO fed to different ends of the bed, an oxygen chemical potential gradient develops along the bed^4,5,12,13^. One end ‘remembers’ the oxygen chemical potential of the most oxidizing gas it encounters (here H_2_O) via high oxygen content; the other remembers the most reducing (here CO). A chemical potential gradient is naturally established along the bed by the cyclic flow of feed gases. Once steady-cycling is reached a H_2_O feed (from the left of Fig. 1) exits the bed over its most reducing end and is essentially completely converted to H_2_, and a subsequent CO feed (from the right) exits over the most oxidizing end and is fully converted to CO_2_. The counter flow produces separated H_2_ and CO_2_ product streams. When operated such that the extremes of the oxygen chemical potential remain within the body of the bed, the reactor can achieve super-equilibrium conversions approaching 100%, well in excess of the 50% achievable under thermodynamic control with a stoichiometric feed at 820 °C (refs. ^2,3^). We have demonstrated stable operation of the memory reactor over thousands of cycles, and how the bed’s memory allows it to switch rapidly between WGS and reverse-WGS operation modes^4,12,13^. Others have since described its use for dry methane reforming to CO_2_ and H_2_ (ref. ^14^).

One of the key operating principles of the reactor is that the oxygen exchange reaction is thermodynamically reversible at each point along the bed. This minimizes thermodynamic losses and is achieved by establishing a continuous and gradual change in oxygen content of the nonstoichiometric ABO_3−*δ*_ OCM. We have previously been able to infer this gradual oxygen content change in a La_0.6_Sr_0.4_FeO_3−*δ*_ (LSF) bed by combining unit-cell parameters measured with operando synchrotron X-ray powder diffraction measurements with thermodynamic models for the oxygen content. There are many assumptions needed in this approach, and it requires knowledge of accurate chemical and thermal expansivity of the OCM under memory reactor conditions and careful in-bed thermometry. In fact, our initial choice of LSF as a proof-of-concept OCM was motivated in part by the availability of the relevant data in the literature^15–20^.

**Fig. 2:**
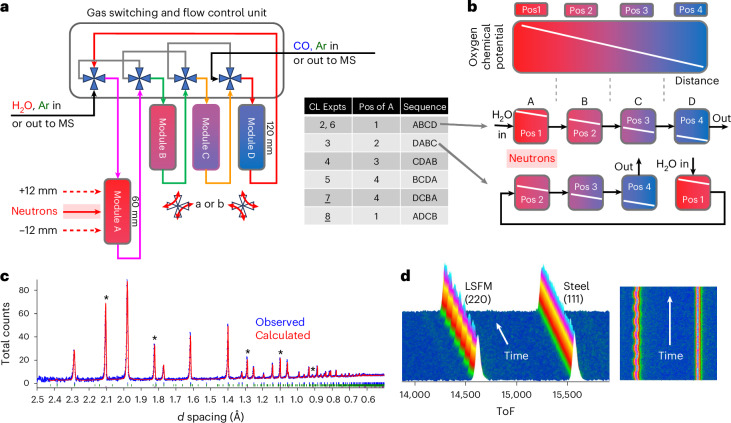
Operando memory reactor. **a**,**b**, The split-bed design in which the gas-switching unit controls the flow direction and sequence of H_2_O, CO and Ar through four linked bed modules A–D (**a**). The output from the overall bed is analyzed by mass spectrometry. The four-way switching valves in settings ‘aaaa’ give bed flow sequence A, B, C, D placing the neutron-monitored bed module A in position 1 of **b**. Neutrons can be directed to the bottom, center or top of module A. The oxygen chemical potential along the single reactor bed of Fig. 1 is split across the four modules (**b**). Gas flow sequences shown in the table place different portions of the overall oxygen chemical potential gradient in the in-beam module A. Underlined experiments in the table use a reverse gas flow relative to that shown in **a**. **c**, Rietveld fit of a 1-min data collection of LSF during a CL cycle at 800 °C. Observed data in blue, calculated in red; vertical tick marks show the allowed positions of LSF and steel sample holder reflections (also marked with a *). **d**, 30-s operando datasets recorded on LSFM colored by count; in each representation the left-hand peak is from LSFM and its position oscillates right and left on reduction and oxidation, the second peak is from the steel sample holder and does not change position on cycling. Data correspond to those shown in Fig. 4d. ToF, time of flight. Source data

Direct measurement of oxygen content using operando X-ray diffraction is extremely challenging. However, Rietveld analysis^21^ of powder neutron diffraction data should be able to provide this information due to the significant scattering contribution from oxygen compared to the metal ions present (neutron scattering lengths 5.803, 8.24, 7.02 and 9.45 fm for O, La, Sr and Fe, respectively). In practice, however, the technical constraints of neutron diffraction experiments are such that either moving the neutron beam along a working bed, or moving the bed relative to the beam, is difficult to achieve. We have overcome this problem through a reactor design that splits the overall OCM bed into multiple modules (Fig. 2a). One module is permanently located in the neutron beam, but by using different gas flow pathways, we can effectively reposition this module so that it sits at different positions along an overall reactor bed (Fig. 2b). This gives us a spatial resolution down to ≲1 cm along a bed of any length. As we discuss below, this has allowed the direct measurement of oxygen content profile in a working proof-of-concept LSF OCM bed. We also show how real-time information can be measured as an OCM bed self-conditions into its operational state, how real-time observation of oxygen content change during CL cycles is possible and how the technique gives information useful for testing the operational limits of new high-capacity OCM materials. Finally, we show initial results on how the CL memory reactor concept can be applied to the important SMR reaction. Here the OCM shows reversible memory effects on both the CL half-cycle and multiple-cycle timescales.

Our split-bed design concept is widely applicable, and could be used for spatially resolved structure-activity profiling on systems such as oxidative dehydrogenation reactions^22^, methane reforming^23^, CO_2_ methanation^24^ and CO_2_ capture and reduction processes^25^, which have only been possible using synchrotron methods. Other systems of interest include automotive emission control, liquid phase oxidation treatments and selective catalytic reduction of NOx.

## Results

### Operando setup concept, design and testing

Obtaining high-quality structural information on a polycrystalline OCM bed material requires: (1) high resolution diffraction data in real space (small Δ*d*/*d*) to give sharp peaks and allow subtle changes in unit-cell parameters and symmetry to be observed; (2) data over a high reciprocal space (*Q*) range to separate effects on diffracted intensity due to static and/or dynamic disorder and site occupancy changes and (3) high intensity to give data with good signal-to-noise ratios, short time resolution and high spatial resolution. Our experiments were therefore carried out on the POLARIS time-of-flight diffractometer at the ISIS pulsed neutron source of the Rutherford Appleton Laboratory^26^.

The fixed experimental geometry of a time-of-flight neutron experiment, the need for an evacuated flight path to reduce background and the finite diffractometer tank size prohibit scanning a working reactor of realistic complexity from a chemical engineering standpoint through the neutron beam to achieve spatial resolution. Our solution to this problem is shown schematically in Fig. 2a, and discussed in more detail in Supplementary Information Sections 1 and 2 (Supplementary Figs. 1–8). The overall reactor bed is broken down into four separate modules. One (Module A) is permanently located inside a furnace mounted on the diffractometer. The remaining three are located in a separate external furnace and connected in series. A gas-switching unit allows the flow of different reactive (H_2_O, CO for WGS) and purge (Ar) gases through the bed modules in different sequences and directions. By flowing gases through the modules in sequence A, B, C, D the in-diffractometer module is in position 1 of Fig. 2b (note that the sequence begins with the module supplied with H_2_O and ends with the module supplied with CO). Sequence D, A, B, C moves module A to position 2, and so on. This allows the neutron beam to probe four different positions along the overall bed. Higher spatial resolution can be achieved in two ways. First, it is possible to use the diffractometer beam jaws to select different ~10 mm vertical portions of the neutron beam. We have shown that this can give high-quality diffraction information at positions centered up to ±12 mm from the central position on POLARIS (Supplementary Fig. 9). It is also possible to use different lengths for the individual external bed modules B–D. By changing both bed flow order and flow direction, a wide variety of spatial locations along the overall bed can be sampled. Bed lengths in ratios of 1:1:2:4 for A:B:C:D would allow coverage of all spatial positions in a working reactor of arbitrary length.

Details of a reactor design exploiting this concept are given in the Supplementary Information. In brief, module A uses a 0.65 mm wall thickness 60 mm 316L stainless steel tube as the in-beam sample holder. Other beds are held in stainless steel tubes in an external furnace and gas flow pathways through heated connecting pipes are controlled by a gas supply and switching unit. This unit communicates its valve state changes to the neutron data collection software and can trigger synchronous data acquisition. The setup uses mass spectrometry to quantify gas evolution from the bed, allowing us to correlate OCM structural changes with the chemical reactions occurring.

Figure 2c shows a powder diffraction pattern of LSF collected at 800 °C in 1 min with this setup. We obtain powder diffraction data of suitable quality for Rietveld analysis on a rapid timescale. In addition to peaks from the OCM, we see peaks of similar intensity from the steel sample can. These can be Rietveld-fitted alongside those of the OCM and, from the measured thermal expansion of the steel, can give a direct measurement of the local bed temperature throughout the experiment; processing details are in the Supplementary Information Section 5. This helps us separate thermally and chemically driven changes in the sample. Figure 2d gives an indication of the time sensitivity of the method, and shows a 3D view of 125 30-s data collections during CL with a La_0.6_Sr_0.4_Fe_0.67_Mn_0.33_O_3−*δ*_ (LSFM) OCM as discussed below. Significant changes in sample peak positions, correlating with structural changes of the bed, are readily observed. The peaks of the sample holder remain essentially unchanged.

### Direct evidence of an oxygen chemical potential gradient

**Fig. 3:**
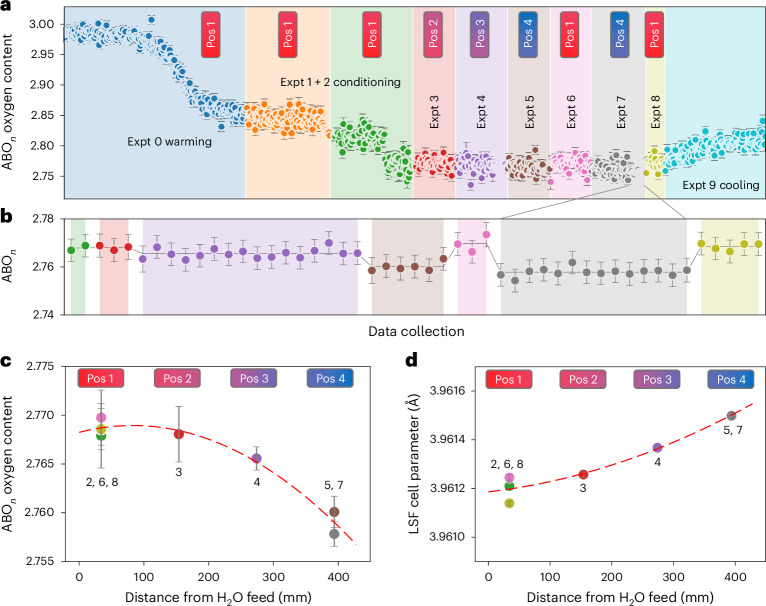
LSF cycling. **a**, Oxygen content of LSF from Rietveld analysis. Rapid data collections were performed as LSF was heated to 800 °C to monitor the gross changes in oxygen content during conditioning with module A in Position 1 of Fig. 2. CL was then performed with different bed positions or position histories in eight separate experiments (different background color shadings). **b**, Oxygen contents from just the longer data collections under Ar at the end of each experiment (Expt.) (for example, the final 14 long collections from experiment 7), the gray line shows the average values. **c**, The average refined oxygen content as a function of module A distance from the oxidizing feed end of the overall bed. **d**, The corresponding unit-cell parameters. Points in **b**–**d** are color coded by experiment number. Points in **a** and **b** are plotted against the data collection number; equivalent plots as a function of total elapsed time are included in Supplementary Fig. 11. Error bars in **b** are derived from the least-squares fitting; error bars in **c** and **d** are the standard error of the mean from the repeat experiments depicted in **b**. Source data

To obtain direct quantitative measurement of oxygen content along the OCM bed of a memory reactor, the reactor of Fig. 2 was configured with a 60-mm LSF bed in the diffractometer bed module A and three 120-mm beds in modules B–D. The total active OCM mass was 84 g. The OCM was loaded in an oxidized La_0.6_Sr_0.4_FeO_3_ state then warmed under Ar to 800 °C with powder patterns recorded in time-resolved slices. CL was then commenced to condition the bed with a flow regime of top, 2 min Ar, 4 min H_2_O and 5 min Ar; bottom 2 min Ar, 4 min CO and 5 min Ar (top and bottom refer to the feed location to module A). These conditions target a gas conversion of 70%, significantly higher than for the mixed-gas reaction. Figure 3a shows the change in bed oxygen content from Rietveld analysis of the corresponding 380 short diffraction patterns recorded (the shaded regions labeled experiments 0–2 ‘warming’ and ‘conditioning’). As expected from previous studies, LSF initially loses oxygen on heating under Ar to give La_0.6_Sr_0.4_FeO_~2.85_. The overall oxygen loss from diffraction agrees well with O_2_ loss from off-line thermogravimetric analysis (Supplementary Fig. 8). On initial gas cycling (experiments 1 and 2), the module A remains relatively oxidized until sufficient reducing gas has flowed to first reduce modules D, C and B, then reduce module A to La_0.6_Sr_0.4_FeO_~2.80_. At this point the bed is conditioned and ready for CL. Supplementary Fig. 10 shows that gas conversions from mass spectrometry follow the expected behaviors during conditioning.

Once steady-cycling was achieved, we recorded a series of longer (>30 min) data collections under Ar flow to give higher-quality structural information and to confirm that the bed state does not change under these conditions (Fig. 3b and Supplementary Fig 12). The bed stability for extended (>7 h) periods confirms minimal oxygen transport occurs via the gas phase under Ar. This is crucial for the reactor retaining its ‘memory’ of reactive gas chemical potentials, and would allow a real-world system to respond to changing throughput by having reactor tubes conditioned and held under Ar ready to go.

Gas flows were then switched to place module A sequentially in positions 2, 3, 4 and 1 with H_2_O flow to the top. CL was performed in each position as rapid powder diffraction data were recorded, followed by a series of long data collections once steady-cycling was reached. We then reversed the gas flow directions by switching the reactive gas feeds and placed module A in positions 4 then 1. We thus obtain equivalent spatial information on the OCM from multiple rig configurations, which helps check for systematic errors.

Figure 3b–d shows key results from Rietveld analysis of the long steady-state data collections at the end of each CL run. Figure 3c plots the Rietveld-refined oxygen content as a function of distance from the H_2_O feed of the bed. As anticipated, we see a smooth decrease in oxygen content, and therefore oxygen chemical potential, from one end of the bed to the other. The trend is well defined despite the small overall oxygen content changes under these operating conditions, and demonstrates the precision (Δ*δ* ≈ ±0.003, 0.1%) in O content achievable from careful operando neutron studies. The consistency between oxygen contents extracted for experiments in which the measured module was at different physical points in the flow path, but is expected to be in an equivalent chemical state, gives confidence in the measurements and analysis methodology. Figure 3d shows equivalent cell-parameter plots, and we see the expected increase in cell parameter as oxygen content decreases and Fe is reduced. The chemical expansivity of ~0.03 Å/Δ*δ* is comparable to previous estimates.

The small cell-parameter changes (~0.0003 Å) in Fig. 3c highlight a key advantage of operando neutron experiments, in that the chemical expansion observed would be comparable to the thermal expansion from a ≲2 K temperature change. In our dynamic experiments, we see temperature changes of a few Kelvin on gas reversal and the OCM bulk takes ~20 min to fully thermally equilibrate after a change in conditions. During this time cell parameters will change (Supplementary Fig. 12). In contrast, Fig. 3b shows that the directly extracted O content is unchanged during this period.

### Time-resolved studies on a high-capacity OCM

LSF was initially chosen to test the concept of the memory reactor for several reasons: (1) the dependence of its oxygen content and unit-cell parameter on *p*(O_2_) and *T* are known; (2) it is stable under WGS conditions and (3) it does not undergo phase transitions under memory reactor steady-cycling conditions, allowing thermodynamic reversibility. One significant drawback is its low oxygen exchange per cycle (Δ*δ* ≲ 0.02 per OCM formula unit). Mn-substituted La_0.6_Sr_0.4_Fe_0.67_Mn_0.33_O_3−*δ*_ (LSFM) potentially allows higher oxygen exchange, and our initial studies using CL operando X-ray diffraction showed Δ*δ* values of 0.05–0.1, around five times higher than LSF^27^. The larger oxygen content changes allowed us to use a single bed module for operando neutron work and looping was performed using CO and CO_2_ for the reducing and oxidizing feeds, respectively, as a proxy for the WGS reaction.

**Fig. 4:**
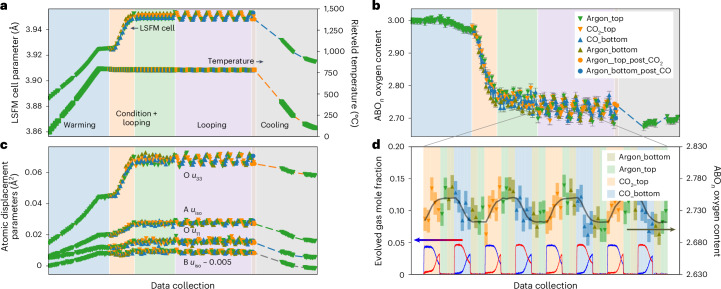
LSFM cycling experiments. Selected results from LSFM cycling experiments on a single bed. **a**, Pseudo-cubic cell parameter of LSFM and sample holder-derived temperature. **b**, Oxygen content. **c**, Atomic displacement parameters; O *u*_11_/*u*_33_ are mean-squared displacement along and perpendicular to the B–O–B link. *u*_iso_ values for B shifted down by 0.005 Å^2^ for visualization as they overlap O *u*_11_ values. **d**, High-precision oxygen content derived from the relationship between unit-cell parameters and oxygen content from the whole experiment (gray line) compared to values extracted from a single Rietveld analysis (points, with uncertainties as shaded bars). The lower plots in **d** show the gases evolved (CO blue, CO_2_ red). Background colors in **a** to **c** represent warming, conditioning, 3× CL then cooling experiments (numbers 10–12 and 14–17 in Supplementary Table 2); in **d** background color represents gas flow, and the total time of the data collections shown is 67 min. Oxidizing gas flow is shown in orange, reducing in blue and Ar purge in green and olive; triangles up and down indicate gas flow directions to the bed. Error bars show the standard uncertainties from the Rietveld refinement. Source data

Figure 4 summarizes key results from Rietveld analysis of data recorded on an initially oxidized bed warmed under Ar from room temperature, brought to steady-cycling conditions, then cycled under different gas flows. We again see a wealth of important information. Figure 4a shows the pseudo-cubic cell parameter through the experiment and the Rietveld-extracted bed temperature. We see a smooth increase in cell on warming due to thermal expansion, followed by a rise of comparable magnitude as CO/CO_2_ cycling decreases the average bed oxygen content to its steady-cycling value of ~2.75 (Fig. 4b). We then see significant and correlated changes in both the cell parameter and oxygen content under cycling. Figure 4a includes the bed temperature derived from the cell parameter of the steel sample holder, which allows us to monitor local temperature changes under different gas flows. The LSFM cell parameter increases under the reducing gas flow even though it is slightly cooler, proving the changes are chemically and not thermally driven.

Figure 4c shows the atomic displacement parameters (anisotropic for oxygen) of OCM atoms. These give an indication of the combined static and dynamic atomic disorder through the experiment and are represented here as mean-squared displacements in Å^2^. These reveal the expected increase in atomic vibrations with temperature on heating, followed by significant additional rises for the A and O sites and a smaller increase for the B site on reduction. The O site shows the most significant increase in the direction perpendicular to the B–O–B bond. These increases reflect the increased local static and dynamic disorder on these sites associated with oxygen vacancy formation. Systematic oscillations are seen in the displacement parameters on cycling, with local disorder increasing on reduction and decreasing on oxidation in line with structural expectations. The ability of neutron diffraction data to provide separate information on oxygen content (from crystallographic site occupancies) and local disorder (from displacement parameters) is a key enabler of these experiments. The neutron data also showed that LSFM remains metrically cubic and is magnetically disordered throughout the CL experiments (Supplementary Fig. 16). This confirms that there are no OCM phase transitions during cycling, which ensures thermodynamic reversibility.

Figure 4d focuses on the third CL experiment performed (the region shaded purple in Fig. 4) and demonstrates the speed at which useful information can be obtained by this operando method. Here, we automatically triggered repeat 30-s data collections on each gas flow change. The triangular data points in Fig. 4d show the Rietveld-derived oxygen content from each 30-s data collection. The solid gray line shows the unit-cell parameter scaled by the empirical relationship between cell and oxygen content discussed below. The lower red and blue traces show the exit gas composition by mass spectrometry. As expected, the scatter in Rietveld-derived oxygen content from a 30-s data collection is significant, but we still see strong correlation between oxygen content and gas flow: as CO_2_ is flowed over the OCM bed we see oxidation, which plateaus under the Ar inert gas flow; we then see reduction under CO flow. This behavior correlates well with our gas analysis.

We can use the information from the whole CL experiment to derive an empirical relationship between the OCM unit-cell parameter (which is well defined by a 30-s data collection) and oxygen content (which is less well defined). This allows us to derive the oxygen content for each 30-s time slice directly from the unit-cell parameter giving the dark gray trace of Fig. 4d (Supplementary Information Section 7). This use of unit-cell parameter to deduce oxygen content is similar to the method that had to be used in previous X-ray experiments on the memory reactor^4,12,13^, but here it is achieved without the need for any previous knowledge of the thermal and chemical expansivity of the OCM.

These experiments also demonstrate the significantly higher oxygen exchange between solid and gas phases for LSFM (Δ*δ* ≈ 0.07 per half-cycle in La_0.6_Sr_0.4_Fe_0.67_Mn_0.33_O_3−*δ*_ versus Δ*δ* ≲ 0.02 for LSF), which allows higher chemical conversion per half-cycle. The diffraction data also show that the oxidation state changes used in these studies are slightly too extreme for this specific OCM, and, for example, drive the development of minor impurity phases of (La,Sr)_2_(Fe,Mn)O_4_ and MnO after ~24 h of CL. The ~2 wt% of impurity formed is insufficient to affect overall conversions on this timescale, but would potentially be important in long-term operation. The early impurity detection enabled by operando methods allows rapid exploration of the operational limits of the reactor.

### Memory reactor SMR

**Fig. 5:**
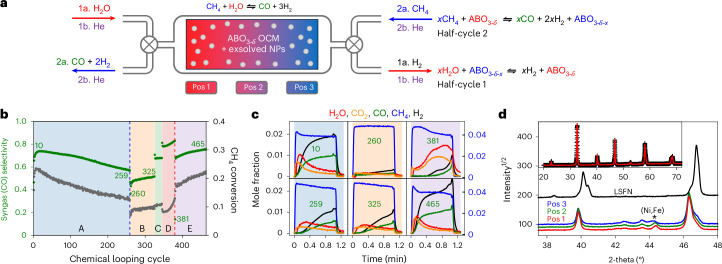
CL-SMR with LSFN. **a**, The CL-SMR process. **b**, Selectivity to syngas (CO + 2H_2_) formation (green points) and CH_4_ conversion (gray points) under different cycling conditions A–E at 750 °C; the blue vertical dashed line shows where the bed was cooled then rewarmed, and the red vertical dashed line where it was reset to optimum conditions by extended oxidation; the total operation time is around 5.5 days. Green numbers correspond to the cycles plotted in **c**. **c**, Gas evolution during 1-min CH_4_ flow for selected cycles; CH_4_ mole fraction is shown on the right-hand axis. **d**, The upper black plot shows the powder pattern of as-synthesized LSFN, and the inset a Rietveld fit using a rhombohedral structural model; lower blue, green and red plots show diffraction patterns from material at Pos 1, Pos 2 and Pos 3 of **a** post experiment. Source data

The need for operando studies is further highlighted by preliminary work to demonstrate that the memory reactor concept can be extended to the SMR reaction (CL-SMR), Fig. 5. In this process CH_4_ is partially oxidized by the OCM to produce syngas (CO + 2H_2_) in one half-cycle, then the bed re-oxidized by H_2_O to produce further H_2_ in a second half-cycle, Fig. 5a. This process needs a more complex OCM capable of methane activation, and we have shown in ref. ^28^ that this can be achieved by exsolution of matrix-stabilized (Ni,Fe) nanoparticles from a La_0.6_Sr_0.4_Fe_0.9_Ni_0.1_O_3_ (LSFN) precursor in an initial catalyst conditioning step. Figure 5b shows that a memory reactor with such a bed can show high selectivity for syngas production over hundreds of cycles, with minimal carbon deposition. Such long-term stability for CL-SMR is rare.

The CL cycles labeled A in Fig. 5b used a preconditioned LSFN OCM bed and a chemistry-balanced CH_4_/H_2_O flow (full details of all conditioning and cycling experiments are in Supplementary Section 8). Within a few cycles of operation, gases evolved in the CH_4_ half-cycle (for example, Fig. 5c cycles 10 and 259) show high selectivity to syngas as demonstrated by the 1:2 CO:H_2_ evolution ratio, and a conversion of around 20%. The complex nature of the multicomponent OCM means that reactor performance can evolve over hundreds of cycles, and, with this combination of preconditioning and operation, the performance gradually decreases. Cycles labeled B, C and D were performed after cooling the bed, holding it at room temperature under helium for several days then reheating. Cycles B show that a reheated bed operated under the same conditions as cycles A gradually recovers the performance expected over around 50 cycles (for example, Fig. 5c cycles 259 and 325 show comparable gas evolution). Cycles C and D show how syngas selectivity and conversion can be adjusted by using different CH_4_/H_2_O flow times. Cycles D used an excess of CH_4_ over H_2_O such that the bed becomes increasingly over-reduced over time. As observed for LSFM, the over-reduction is associated with the formation of new perovskite-related phases such as (La,Sr)_2_FeO_4_ and (La,Sr)_3_Fe_2_O_7_. We find that this deliberately over-reduced bed can be reset to its original performance by an extended oxidative treatment under H_2_O that regenerates LSFN (as indicated by the vertical dashed red line between cycles D and E in Fig. 5b). After this treatment, the gases evolved in the CH_4_ half-cycle (Fig. 5c cycles 381 and 465) show a steady increase in selectivity to syngas production over a few tens of cycles as the bed reconditions and an optimum oxygen gradient and metal particle content is re-established. Performance at the end of the experiment (cycle 465) actually exceeds that of the initial bed under the same feed conditions. Figure 5d shows diffraction patterns of the OCM from various points along the bed at the end of the experiment. These show significant peak shifts relative to the as-synthesized oxidized LSFN as expected, and subtle changes in the relative quantities of exsolved metal particles and perovskite phases. Bed memory effects both in-cycle and over multiple cycles mean that the type of neutron operando experiments described above will be crucial in fully understanding and optimizing this system.

## Discussion

We have demonstrated that it is possible to build and operate a working memory reactor under conditions where the whole OCM bed can be monitored by operando neutron diffraction. This gives valuable insight into the atomic-level responses of the OCM as it functions. Our setup allows both spatial (down to ≲1 cm) and temporal (down to ≲30 s here) resolution of a realistically scaled fully operational and automated reactor. From the data quality, we estimate useful information could be obtained down to ~10 s. Achieving spatially and time-resolved information of direct relevance to an important chemical engineering transformation by neutron scattering is an important advance.

For the proof-of-concept LSF OCM, the multi-bed spatially resolved design has given direct observation of the development of a smooth gradient in oxygen content, and therefore oxygen chemical potential, along the bed. This could be reliably proved despite the small oxygen changes expected for this material, and is an important milestone in showing how these reversible reactors work.

We have also been able to study LSFM, which has an oxygen transfer capacity many times higher than LSF. Here we successfully track the OCM’s evolution on a timescale significantly shorter than a CL half-cycle. We can monitor the OCM state under reactive gas flow in real time, which allows the rapid screening of bed performance under different reaction conditions. This enables the early observation of minor bed degradation. Without this operando approach such information can only be achieved through expensive (in terms of OCM usage and time) batch experiments. The sensitivity of neutron diffraction to oxygen content also lets us use high-precision cell-parameter measurements to infer oxygen content from extremely fast neutron diffraction experiments without any previous knowledge of the thermal or chemical expansivity of the OCM under the reactor conditions.

Finally, we have shown how the memory reactor concept can be extended to the SMR reaction, potentially allowing distributed and scalable production of syngas or coupling of SMR and WGS memory reactors. The complexity of the bed chemistry, and the different timescales on which it displays memory effects, mean that operando experiments of the type described here will play an essential role in process optimization.

Overall, these operando neutron measurements provide crucial and otherwise inaccessible information on the functional behavior of memory reactors. More generally, the work shows the power of operando neutron experiments for optimizing complex chemical processes, and our split-bed concept means that this can be done on a scale that is relevant to real-world chemical engineering challenges.

## Methods

### Setup design and operation

An ISIS in situ stainless steel cell (60 mm length) was used for Module A inside the POLARIS diffractometer. This was installed within the standard ISIS one-quarter-inch (roughly 6 mm) reaction cell assembly and loaded inside a vanadium RAL vacuum furnace (Supplementary Fig. 1). Pre- and postbed reactors were fabricated from half-inch (1.3 cm) outer diameter stainless steel tubes of 900 mm in length. Standard Swagelok VCR compression fittings were used at both ends of each reactor and kept external to the furnace heated zone. The three reactors were held in a triangular arrangement and equally spaced within a single vertical tube furnace (Carbolite-Gero TS3 12/60/600). The four reactors, including module A, were connected in series with heat-traced one-quarter inch gas transfer lines. Four-way crossover valves were used to select the gas feeds, control CL cycling, enable outlet gas analysis with mass spectrometry and set the order of gas flow through the beds to give spatial resolution. Further details of operation and mass-spectrometry calibration are provided in Supplementary Information Section 1.

### Sample preparation, CL protocols, neutron methods and Rietveld analysis

Details of the OCM bed materials used, their characterization and bed packing are given in Supplementary Information Section 2. Neutron powder diffraction data were collected on the POLARIS diffractometer^26^ at the ISIS neutron and muon facility of the Rutherford Appleton Laboratory. POLARIS receives a high neutron flux with energies giving access to *d* spacings from 0.2 to 20 Å across its detector banks. The high detector coverage (5.67 sr of solid angle) gives the fast count rates required for this experiment^26^.

A similar experimental protocol was followed for LSF and LSFM. The sample was warmed from room temperature to a furnace set temperature of 800 °C under Ar, with data collected in time-resolved segments. Sample heating was continued during short periods of beam outage to ensure a constant heating rate. External beds were heated at a comparable rate. After a hold period at 800 °C, CL was initiated. Rapid data collections were automatically triggered by the CL rig either for fixed time periods during specific gas flows (LSF), or when the rig valve state switched. After each CL run the LSF bed was held under a flow of Ar and longer data collections recorded to monitor any OCM evolution with time, and give higher-quality structural information. Full details are given in the Supplementary Information.

Rietveld refinement was performed using TOPAS Academic v.7 (refs. ^21,29^). All five detector banks were simultaneously fitted using structural models for the OCM and for the steel sample cell. Instrumental calibration constants and peak shape contributions were determined using a Si standard. Sample and holder peak shapes were fitted by convoluting terms describing size and strain broadening. Backgrounds were fitted using a Chebyshev polynomial. Both LSF and LSFM undergo structural and magnetic phase transitions under the range of temperature and oxygen partial pressure conditions studied. To allow the consistent structural model over all datasets needed to reliably follow oxygen changes, the OCM structure was described using a $R\bar{3}r$ rhombohedral model, with the single structural degree of freedom describing oxygen tilts allowed to refine along with the oxygen site occupancy. The magnetic contributions to the diffraction at lower temperatures were described using a separate magnetic phase with Shubnikov group 167.103 (unified symbol $R\bar{3}c.1[R\bar{3}m]$) and moment along the [111] axis of the rhombohedral cell^30^. Isotropic temperature factors were used for A and B sites, and anisotropic for O. The O anisotropic displacement parameter was constrained to have its axis of rotation along B–B directions to mimic the degrees of freedom in a cubic model. Each refinement used a total of 119 parameters to fit all five banks. For detailed analysis of CL experiments at high temperature a cubic model was used with two fewer parameters. Least-squares correlations between oxygen content and other parameters were low (<35%). Data from Rietveld refinements were automatically processed and linked with metadata describing the rig gas flow state and mass-spectrometry output using Python scripts. The unit-cell parameter of the steel sample holder was measured during warming the LSF bed, and the empirical thermal expansion used as a measure of the local bed temperature changes under gas flow. As module A has a gas preheater only on its top inlet, the sample is slightly cooler (~7 K) under gas flow from the bottom and we see minor fluctuations in the sample holder-derived temperature during CL.

Full details of the CL-SMR reactions are given in the Supplementary Information Section 8.

## Supplementary information

Supplementary Information Supplementary figures, tables and discussion.

Supplementary Data 1 Experimental information.

Supplementary Data 2 Rietveld analysis information.

Supplementary Data 3 Rietveld analysis information.

Supplementary Data 4 Rietveld analysis information.

Supplementary Data 5 Conversion information.

## Source data

Source Data Fig. 2 Statistical source data.

Source Data Fig. 3 Statistical source data.

Source Data Fig. 4 Statistical source data.

Source Data Fig. 5 Statistical source data.

## Data Availability

All data that support our findings are available within the text or the Supplementary Information. Neutron scattering data are available at 10.5286/ISIS.E.RB2220074-1 (ref. ^31^). Source data are provided with this paper.
