# Supplementary material for: Probing dynamic oxygen exchange for hydrogen production with operando neutron diffraction
Source: Nat Chem Eng. 2025 Jun 4;2(7):447–55. doi: 10.1038/s44286-025-00231-9 (PMC12283380; doi:10.1038/s44286-025-00231-9)
Supplement: Supplementary file 1 — Supplementary figures, tables and discussion. [file 44286_2025_231_MOESM1_ESM.pdf]

nature chemical engineering

Supplementary information https://doi.org/10.1038/s44286-025-00231-9

Probing dynamic oxygen exchange for hydrogen production with operando neutron diffraction

In the format provided by the authors and unedited

Supplementary Information Table of Contents

1. Operando neutron powder diffraction chemical looping rig design 1
Reactor modules design and loading 1
Gas flow control system 1
Experimental rig mounted on beamline 3
2. OCM preparation and testing 4
LSF ($La_{0.6}Sr_{0.4}FeO_{3-\delta}$) granules 4
LSFM ($La_{0.6}Sr_{0.4}Fe_{0.67}Mn_{0.33}O_{3-\delta}$) powder 6
LSFN ($La_{0.6}Sr_{0.4}Fe_{0.9}Ni_{0.1}O_{3-\delta}$) powder 6
3. Beam offset tests of Polaris spatial resolution 6
4. LSF and LSFM operando CL experiments 8
5. Operando CL Rietveld refinement results 9
6. LSF operando CL experiments 10
Gas flows 10
Gas conversion 10
Rietveld-derived parameters 12
7. LSFM operando CL experiments 14
Gas flows and conversion 14
Dependence of oxygen content on unit-cell parameters 14
8. LSFN CL-SMR demonstration experiments 15
9. Supplementary references 17

1. Operando neutron powder diffraction chemical looping rig design

Reactor modules design and loading

The in-beam reactor module A used an ISIS 316L stainless steel sample cell. Its design and position on the ISIS reactor cell mounting stick is shown in Fig. 1. The overall tube length is 60 mm with an inner/outer diameter of 10.95/11.30 mm. A P1 quartz frit was used to support the sample. Modules B–C were made from inner/outer diameter 10.21/12.7 mm stainless steel tubes. The active LSF material (120 mm) was centred in the tubes between alumina packing material (alumina balls, Saint-Gobain NorPro, Denstone®, 99.99% α-alumina, 1-2 mm size) supported on steel gauzes. The modules were held in a vertically mounted Carbolite-Gero TS 12/60/600 three zone furnace. For LSF experiments masses used in bed modules A, B, C and D were 15.23, 23.03, 23.01 and 23.03 g, respectively.

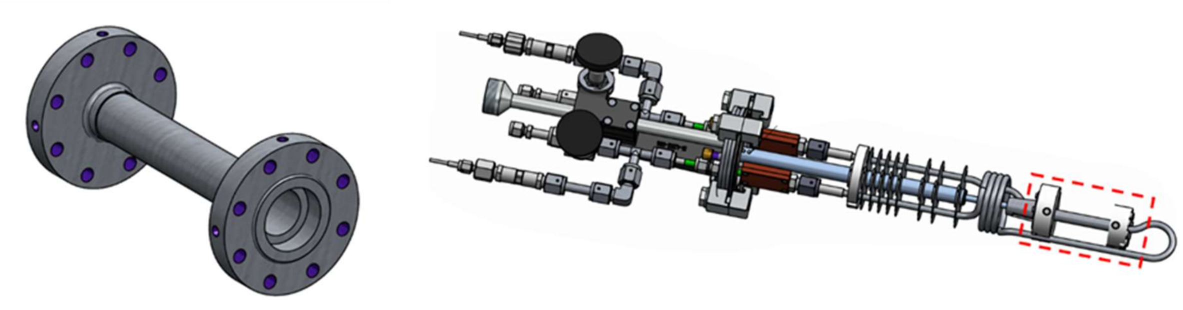

Fig. 1 | Standard ISIS in situ cell and reaction cell assembly. The dashed red box shows the position of module A within the furnace centre-stick assembly.

Gas flow control system

The gas flow control and bed switching systems were designed so that chemical looping (CL) could be performed with either a single reactor module (as for LSFM experiments) or using the four-module design of Fig. 2 in the main text. The single reactor gas flow control system is shown in Fig. 2. The bed module switching unit is shown in Fig. 3 and sits between V3 and the reactor(s). Gases were supplied by BOC. Water was supplied by a bespoke delivery system built by IGI systems. Water from

a feed tank was fed to a heated vapouriser block using a rotary pump (Tuthill) and Coriolis meter (Bronkhorst M120) then mixed with Ar. Delivered water content concentration was verified using a chilled mirror hygrometer (Alpha Moisture System).

Prior to commencing CL the gas flow system was typically configured according to Fig. 2, with an Ar flow to the top of the bed. This ensured that stable gas flows were established prior to experiments and minimal disruption to the system occurred on commencing cycling. To initiate a H_2O half cycle, V2 was switched automatically from position B to position A to direct H_2O to the top of the reactors. When the half cycle was finished, V2 was switched back to position B to re-introduce Ar to the top of the reactors for purging H_2. For the subsequent CO half cycle, V3 was initially switched to direct Ar to the bottom of the reactors, then V1 was switched to position A to introduce CO. This was then followed by an Ar purge by switching V1 back to position B.

Details of the gas flow rates, times for specific experiments and gas valve states for specific experiments are given later in SI sections 6 and 7.

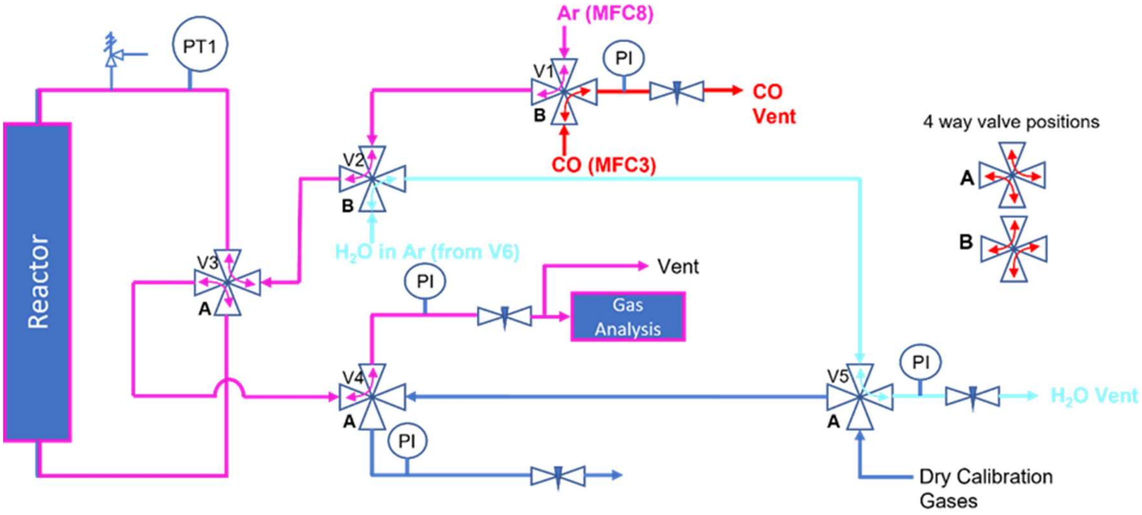

Fig. 2 | Flow diagram and valve states at the start of chemical looping experiments. V1 is switched when a CO half cycle is performed. V2 is switched when a water half cycle is performed. V3 is switched to control the direction of flow through the reactor modules. Note, the single reactor shown here also represents the four modules in series, which are connected via additional four-way crossover valves. PI = pressure indicator; PT = pressure transducer.

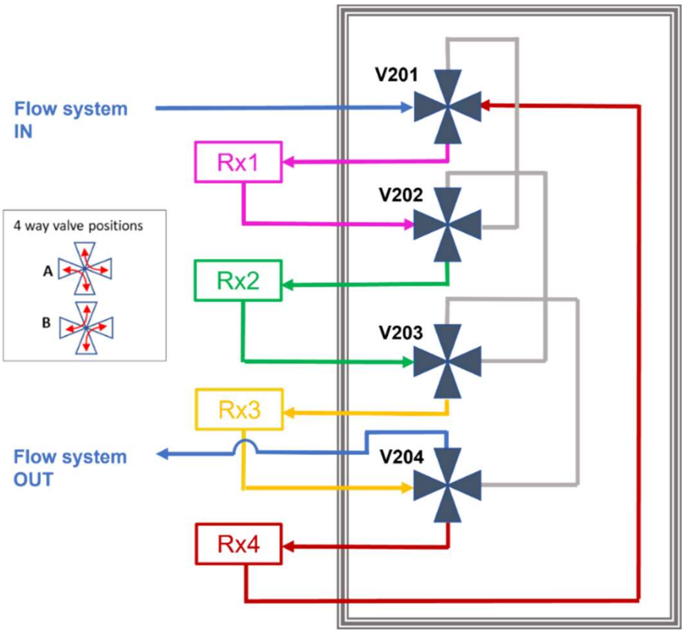

Fig. 3 | Bed switching unit. Schematic of the gas switching unit used to change reactor module order in the LSF experiments. Rx1 (Module A) is permanently in the POLARIS furnace and mounted in the beam. Rx2 to Rx4 (Modules B–D) are in an external tube furnace. V3 of the previous figure is connected to the switching unit at V201. Positions of V201 to V204 determine the effective position of Module A in the reactor sequence. The exit gases are returned to the main system by connecting V204 back to V3. The switching valves are located in a heated oven, shown by the black outline.

Experimental rig mounted on beamline

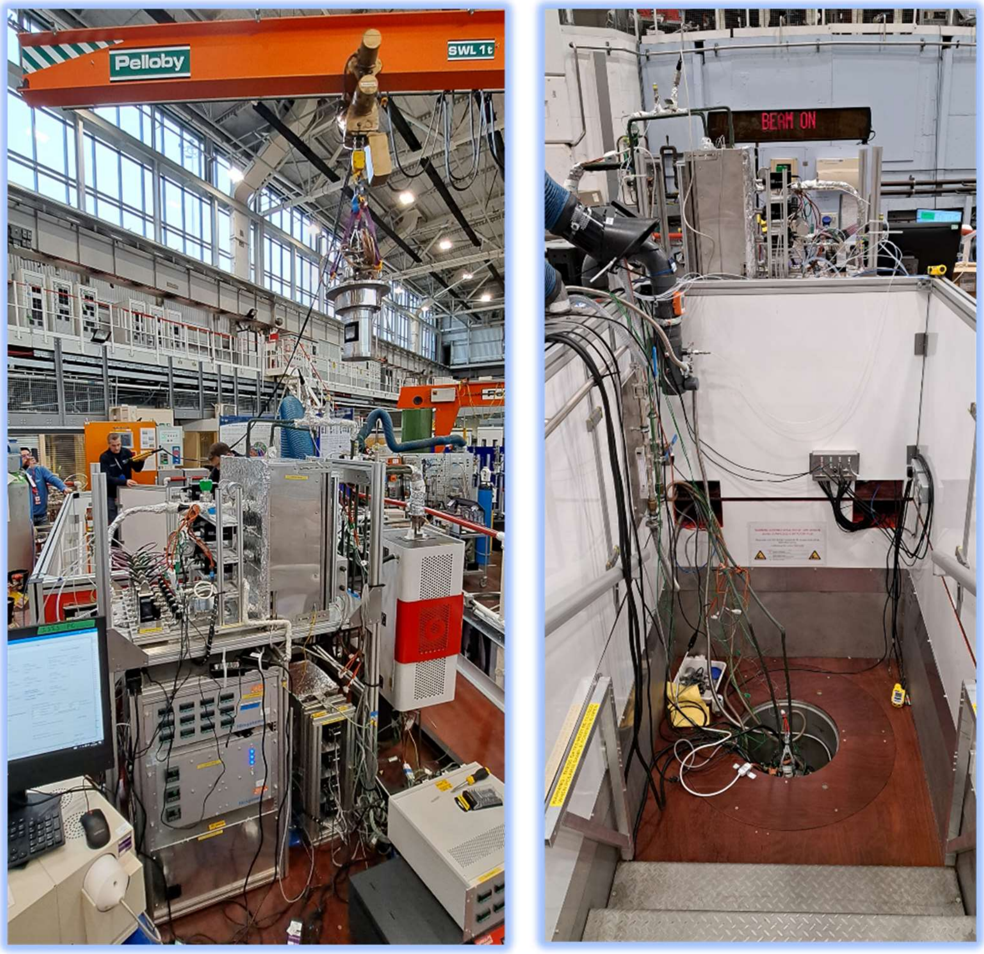

Fig. 4 | Photographs of the experimental set-up. Left: chemical looping flow system (lower centre of picture) and pre- and post- bed furnace (cream and red unit on right hand side). RAL vacuum furnace and *operando* cell assembly (Module A) are being loaded overhead by crane. Right: heat traced transfer lines to/from module A inside the POLARIS vacuum tank (circular opening). Transfer lines pass through the left opening in the rear wall of the sample area to the gas flow and bed switching unit on the platform.

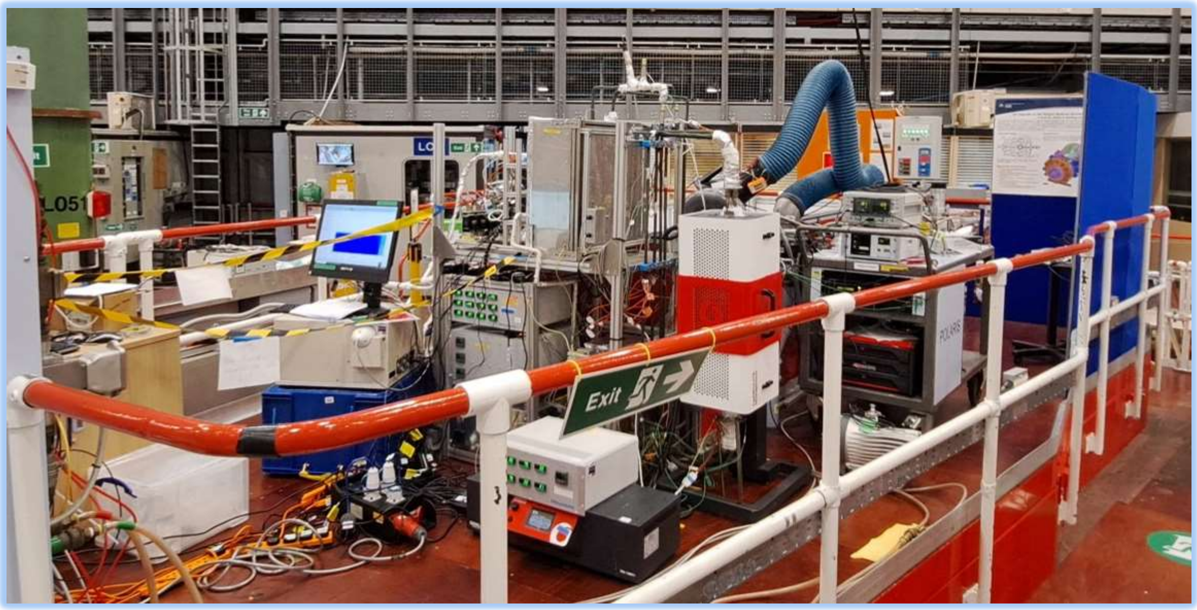

Fig. 5 | Experimental set-up. Photograph of the complete experimental out-of-beam apparatus installed on the POLARIS platform. The diffractometer itself is underneath the platform.

Gas analysis

The outlet gas from the reactor apparatus (centre of Fig. 2 schematic) was analysed by mass spectrometry using a Hiden QGA. The mass spectrometer continually sampled a small portion of exit gases under vacuum (approximately 20 ml/min, STP) for real-time analysis (1-2 measurements per second). The capillary was heat traced, and the inlet fitted with a small cartridge heater to avoid water vapour condensation. A secondary electron multiplier (SEM) detector was used to allow high acquisition rates.

The mass spectrometer was calibrated by flowing each gas of interest (Ar, CO, CO_2, H_2 and water vapour) in turn until a stable signal was achieved for at least 30 minutes. A zero-point reading was obtained where only Ar was fed. Calibration factors were calculated based on the known composition of the gas bottles (nominally 5.0 mol%) and the theoretical water concentration delivered (5.02 mol%). Fragmentation of CO_2 to CO from secondary ionisation was accounted for using a splitting factor of 0.18. Water fragmentation to hydrogen was corrected for with a splitting factor of 0.02. The calibration procedure was repeated at multiple points throughout the experiment to allow signal drift (from mass spectrometer vacuum performance and relative sensitivities to each gas) to be corrected. A linear interpolation between two calibration points was performed.

The mole fraction of each gas at a given time was calculated by dividing the corrected signal by the sum of all the corrected signals present. The mole fraction for each gas was then integrated over each full cycle using MATLAB scripting routines.

2. OCM preparation and testing

LSF ($La_{0.6}Sr_{0.4}FeO_{3-\delta}$) granules

LSF was purchased from PI-KEM and was synthesised by a combustion spray pyrolysis method. Particle size was d_{50} < 1 µm, d_{95} < 5 µm, BET surface area 5–7 m^2/g. It was characterised by powder X-ray diffraction, TGA and ICP analysis. ICP gave a La:Sr:Fe ratio of 0.59:0.41:1.00. Oxygen content was determined by iodometric titration and gave an O content of 3.01±0.01.

Approximately 1 kg was processed in thirty-three 28 g batches to form granules by: (1) preparation of a wet mass with 12.5 wt% water content; (2) Extrusion at 75 rpm using a Caleva MultiLab unit; (3) Oven drying at 75 °C for 30 minutes; (4) Spheronisation at 525 rpm for 3 minutes; (5) sintering in air for 2 hours at 900 °C. Images from different stages of the process are shown below.

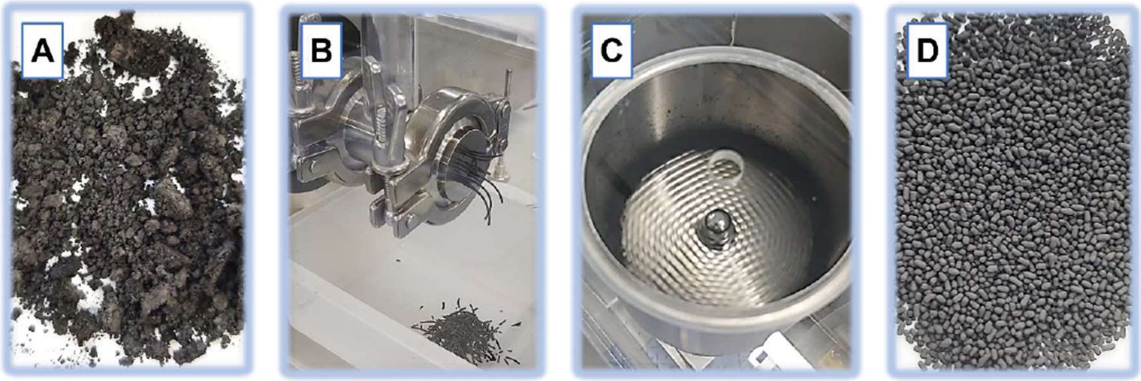

Fig. 6 | Extrusion-spheronisation of LSF. (A) wet mass after mixing with water, (B) extrusion, (C) spheronisation, (D) spheronisation product. Spheronisation conditions in C were optimised to maximise yield in the 700 to 1200 μm range.

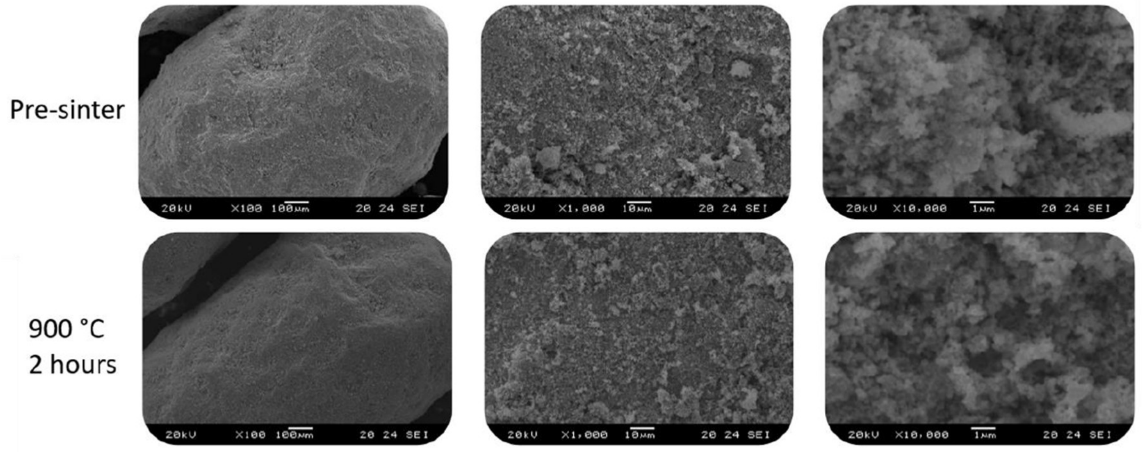

Fig. 7 | SEM images of spheronised LSF. SEM images recorded on spheronised LSF at relative magnifications of ×1; ×10; ×100 before and after sintering in air for 2 hours at 900 °C. Data recorded using a JEOL JSM5600-LV microscope without particle coating.

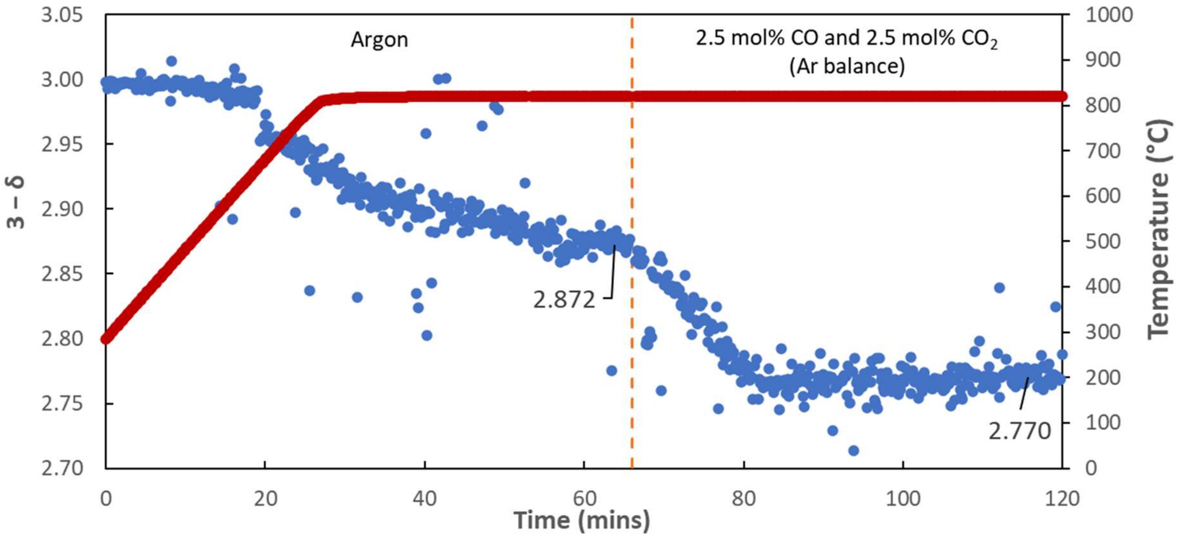

Fig. 8 | TGA of LSF. Sample was initially heated to 820 °C under Ar, then reduced under a 1:1 $CO:CO_2$ buffer gas. Temperature is shown in red and oxygen content as blue points. Data recorded using a Rubotherm TGA.

LSFM ($La_{0.6}Sr_{0.4}Fe_{0.67}Mn_{0.33}O_{3-\delta}$) powder

An oxidised sample of $La_{0.6}Sr_{0.4}Fe_{0.67}Mn_{0.33}O_3$ was synthesised using a modified Pechini method.[1] The metal nitrate precursors, $La(NO_3)_3{\cdot}6H_2O$ (99.9%, 23.43 g), $Sr(NO_3)_2$ (99.0%, 7.71 g), and $Mn(NO_3)_2{\cdot}4H_2O$ (99%, 7.55 g) were weighed and citric acid (CA, 37.98 g) added to give a molar ratio of 1:1 (metal ions:CA). Ethylene glycol (EG, 13.43 g) was added in a ratio of 1.2:1 (EG:CA) and all reagents were subsequently dissolved in deionised water. The resultant solution was poured slowly into a 10 L beaker to provide a large area for evaporation. The beaker was placed into an oven at 80 °C overnight. The resultant cake was gently crushed using a mortar and pestle before calcination (10 °C/min ramp rate) under static air at 1200 °C for 10 hours. The final product powders were ground in a mortar and pestle and sample purity confirmed by powder X-ray diffraction.

LSFN ($La_{0.6}Sr_{0.4}Fe_{0.9}Ni_{0.1}O_{3-\delta}$) powder

An oxidised sample of $La_{0.6}Sr_{0.4}Fe_{0.9}Ni_{0.1}O_3$ was synthesised using an analogous method to that used for LSFM, except that a stoichiometric amount of $Ni(NO_3)_2{\cdot}9H_2O$ was used instead of $Mn(NO_3)_2{\cdot}4H_2O$ as one of the precursors. Its purity and rhombohedral structure were confirmed by powder X-ray diffraction. The resulting powder was sieved to 80–160 μm and subsequently reduced in a flow of 5% H_2 in Ar at 800 °C for 5 hours before being used for chemical looping steam methane reforming.

3. Beam offset tests of Polaris spatial resolution

The potential to achieve higher spatial resolution by offsetting the neutron beam was demonstrated on an LSF sample mounted in module A at room temperature. A 10 mm high by 15 mm wide beam was selected using the diffractometer jaws, and its centre displaced relative to the ideal beam centre over a –13.5 to +13.5 mm vertical range. Diffraction patterns were recorded for equivalent times at each vertical beam height, and data from the highest resolution bank are shown in Fig. 9. Results of Rietveld analysis to show the impact on important structural parameters are included in Table 1. Whilst we see minor changes in overall intensity due to the beam flux profile, there is no significant degradation in resolution (peaks remain sharp) and extracted structural parameters show no systematic variation.

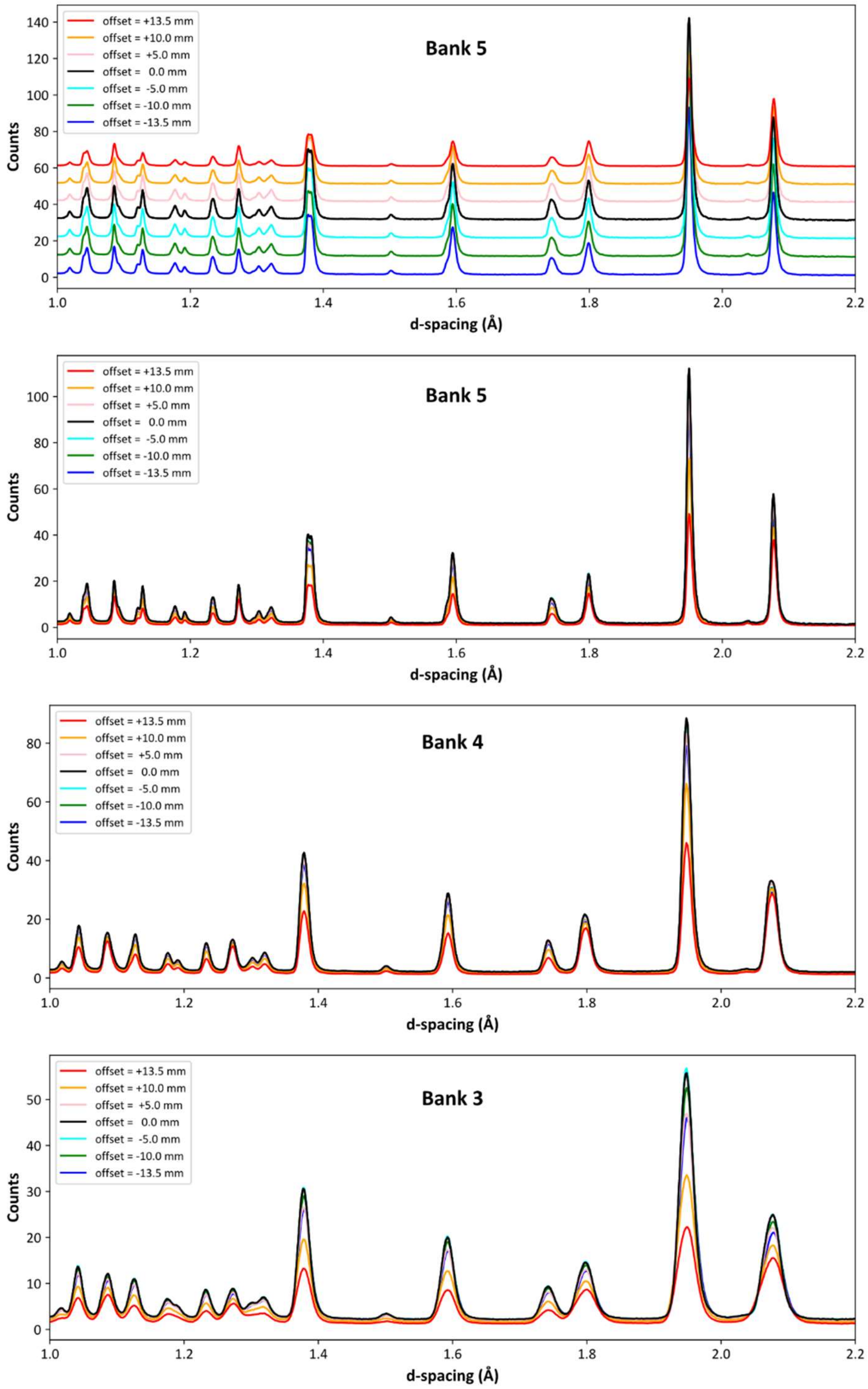

Fig. 9 | Beam offset tests on LSF in stainless steel cell. Powder diffraction patterns of LSF in a steel sample can measured on POLARIS as a 10 mm high by 15 mm wide beam is offset vertically by moving the diffractometer jaws. Highest resolution bank 5 data are shown both superimposed and offset vertically by 10 counts per pattern. Peaks at 2.08, 1.80, 1.27, 1.08, 1.04 Å are from the steel can, all other peaks are from LSF.

Table 1 | Rietveld analysis of beam offset tests on LSF in stainless steel cell. Quantities extracted from a Rietveld analysis of POLARIS bank 5 data collected at different vertical beam offsets. Unit-cell parameters and oxygen site occupancies show no significant changes with the beam at different vertical positions.

Neutron beam centre position (mm)	R_{wp} (%)	Goodness of fit	Lattice parameters (Å)	Unit cell volume (Å^3)	O1 site fractional occupancies	Magnetic moment (μ_B)	Bank 5 scale factor
+13.5	3.68	3.87	*a* = *b* = 5.5266(5)<br>*c* = 13.4475(13)	355.70(7)	1.001(2)	0.98(4)	0.204(3)
+10	3.36	2.84	*a* = *b* = 5.5284(4)<br>*c* = 13.4495(11)	355.99(6)	1.0003(18)	0.97(3)	0.296(3)
+5	3.19	2.98	*a* = *b* = 5.5274(4)<br>*c* = 13.4458(10)	355.76(6)	1.0007(17)	0.92(3)	0.390(4)
0	3.34	3.23	*a* = *b* = 5.5275(4)<br>*c* = 13.4463(10)	355.79(6)	1.0000(17)	0.94(3)	0.431(4)
−5	3.46	3.31	*a* = *b* = 5.5279(4)<br>*c* = 13.4474(10)	355.87(6)	1.0006(18)	0.92(3)	0.423(4)
−10	3.35	3.17	*a* = *b* = 5.5279(4)<br>*c* = 13.4474(10)	355.87(6)	1.0004(17)	0.93(3)	0.408(4)
−13.5	3.19	4.14	*a* = *b* = 5.5273(4)<br>*c* = 13.4461(10)	355.76(6)	0.9995(17)	0.96(3)	0.374(4)

4. LSF and LSFM operando CL experiments

Table 2 summarises experiments (expt) 0 to 17 performed with LSF and LSFM as the OCM as the bed(s) were heated, conditioned, used for CL then cooled. Colour coding matches backgrounds on graphs throughout the paper and Supplementary Information. Where not specified, LSF CL cycles were top: 2 minutes Ar, 4 minutes H_2O, 5 minutes Ar; bottom: 2 minutes Ar, 4 minutes CO, 5 minutes Ar. Full details are given in the SI file “logbook_SI.xlsx”. “Pos” refers to the position of module A as in Fig. 1 of the main text; 1–4 are used for gas flow to the top of module A and 8–5 for gas flow to the bottom of module A.

Table 2 | Operando experiments

Sample	Experiment	Experiment description	Pos	Run number(s)
LSF	N/A	Long data collection before CL experiments at 20 °C	1	135893
LSF	expt0000	Warm to 800 °C over ~4 hours collecting data in 1 min time slices, hold at 800 °C	1	135894-136092
LSF	expt0001	5 × CL cycles then 5 × long data collections	1	136093-136176 then 136176-136185
LSF	expt0002	5 × CL cycles then 2 × long data collections	1	136190-136274 then 136275-136276
LSF	expt0003	10 × CL cycles then 3 × long data collections	2	136279-136320 then 136321-136323
LSF	expt0004	10 × CL cycles then 15 × long data collections	3	136234-136365 then 136366-136380
LSF	expt0005	10 × CL cycles then 5 × long data collections	4	136381-136422 then 136423-136427
LSF	expt0006	10 × CL cycles then 3 × long data collections	1	136428-136469 then 136470-136472
LSF	expt0007	10 × CL cycles then 14 × long data collections	8	136473-136518 then 136519-136532
LSF	expt0008	5 × (1 × CL cycle then 72-min data collection)	5	136533-136553
LSF	expt0009	Cool from 800 °C collecting data in 1 min time slices	5	136554-136664

LSFM	expt0010	Warm to 800 °C over ~6 hours collecting data in 4 min time slices, hold at 800 °C	N/A	136665-136767
LSFM	expt0011	10 × CL (2 min CO, Ar, CO_2, Ar). 1 min data collections. 400 ml/min gas flow. Then 2 × 30 min data collections	N/A	1136769-136810
LSFM	expt0012	11 × CL (2.16 min CO, Ar, CO_2, Ar). 2 × 30 s data collections under reactive gas; 1 × 60 s under Ar. 450 ml/min gas flow. Then 1 × 30 min data collection	N/A	136813-136879
LSFM	expt0013	Test experiments on POLARIS-rig communication protocols, data not analysed.	N/A	136894-1137021
LSFM	expt0014	7 × CL cycles (4.32 min CO_2, Ar_top then Ar_bottom, CO, Ar_bottom then Ar_top) multiple 30 s data collections triggered by valve changes (5 for reactive gases, 4 for Ar). 225 ml/min gas flow (same target conversion as expt0012).	N/A	136894-137021
LSFM	expt0015	2 × CL: 2 min data collection under reactive gas, 30 min under Ar. Sequence: Ar_top, CO_bottom, CO2_top, Ar_top, CO_bottom, Ar_top. No data for final two runs.	N/A	137022-137029
LSFM	expt0016	5 × CL with unbalanced gas flow. 4 min CO_2, 2 min CO. Both gas flows 500 ml/min. Ended on CO cycle. Beam outages mean no neutron data recorded.	N/A	None
LSFM	expt0017	Cool collecting data in 4 min time slices. Beam outages mean not all files contain diffraction data.	N/A	137030-137129

The valve states needed for the gas flow directions for experiments 2 to 8 can be understood with respect to Fig. 2a of the main paper. We will label valve positions as a and b for clarity. Experiments 2 and 6 used a switching unit valve position sequence aaaa (for the 4-way valves from left to right as shown in Fig. 2a) to send H_2O flow along the pink, green, yellow then red pathway of Fig. 2a in the arrowed direction. H_2O therefore enters the top of each module in module sequence ABCD before exiting through the grey pathway to the mass spectrometer for analysis. The CO counterflow uses the same switching module valve positions so CO followed the grey pathway before passing through red, yellow, green and pink pathways against their arrowed directions. It therefore flowed through modules DCBA from each module's bottom. Experiment 3 had valve sequence baab to give H_2O flow sequence DABC (H_2O flowed through grey then red, pink, green, yellow pathways). Experiment 4 had baba to give CDAB and experiment 5 had bbaa to give BCDA. Experiments 7 and 8 swapped the H_2O and CO feeds relative to Fig. 2a. Experiment 7 achieved H_2O flow DCBA by valve settings aaaa and Experiment 8 used bbaa.

The valve positions for both the switching unit (Fig. 3, which is equivalent to Fig. 2a but with a different set of notations) and main flow system (Fig. 2) were set automatically and their values recorded as a valve state code. These are recorded in the electronic logbook logbook_SI.xlsx.

5. Operando CL Rietveld refinement results

Details of the protocol used for Rietveld refinements are given in the methods sections of the article. Parameters extracted by Rietveld refinement are tabulated in the following files:

Table 3 | Data files with Rietveld-extracted parameters

Model	Filename
LSF rhombohedral model used to determine oxygen content, cell distortion and magnetism throughout the whole experiment	lsf_rhomb_results_07_all_processed.csv
LSF cubic model used for high temperature data after bed conditioning	lsf_cubic_results_11_long_all_processed.csv
LSFM rhombohedral model used to determine oxygen content, cell distortion and magnetism throughout the whole experiment	lsfm_rhomb_results_08_all_processed.csv

Cell parameters of the steel can were extracted by Rietveld refinement against data collected during experiment expt0000 as LSF was heated from room temperature to 800 °C then held at this temperature prior to chemical looping studies. Cell parameters were fitted using the expression $a(Fe) = aT^2 + bT + c$ Å with $a = 8.096 \times 10^{-9}$ Å^{-2}, $b = 6.305 \times 10^{-6}$ Å^{-1}, $c = 3.5923$ Å. A linear thermal expansion coefficient of 7.43×10^{-5} K^{-1} was extracted from data from 600 to 800 °C and used for temperature corrections for operando experiments. LSF cell parameters are in excellent agreement with those determined under comparable conditions by previous synchrotron and neutron powder diffraction studies.

6. LSF operando CL experiments

Gas flows

Details of the gas flows and times for the LSF experiments are given in Table 4. A flow rate of 750 ml/min was used throughout as this was the lowest stable flow that could be delivered by the water vaporiser system. A liquid water flow of 1.91 g/hr was used.

Table 4 | Gas flow details for LSF experiments.

Phase	Gases	Flow rate (mL/min, NTP)	Gas Flow Direction	Duration (min)
Pre-oxidation inert	Argon	750	↓	2
Oxidation	5% H_2O in argon balance	750	↓	4
Post-oxidation inert	Argon	750	↓	5
Pre-reduction inert	Argon	750	↑	2
Reduction	5% CO in argon balance	750	↑	4
Post-reduction inert	Argon	750	↑	5

Gas conversion

Gas conversions throughout the LSF CL experiments are shown in Fig. 10 and summarised in Table 5. The bed is initially loaded in an oxidised state and has a composition of around $La_{0.6}Sr_{0.4}FeO_{\sim2.85}$ at the start of expt0001. It therefore shows high CO to CO_2 conversion and low H_2O to H_2 on initial cycling. As the overall bed reaches a composition around $La_{0.6}Sr_{0.4}FeO_{\sim2.80}$ under steady-cycling, conversions reach around 70% for both half reactions. These match the conversions targeted for the *operando* studies. Higher conversions (>95%) can be achieved with shorter gas flows.

When reactor module sequence is changed between different experiments (i.e. CL cycles 21, 31, 41, 51 and 61 of Fig. 10), a lower initial conversion of H_2O is observed (and to a lesser extent CO conversion), which quickly recovers in subsequent cycles. This is expected and can be explained as follows.

In cycles 21, 31, 41 and 61 the most reduced module was moved to the most oxidised position in the sequence. For example, in Cycle 21 the bed sequence changed from DABC (from most oxidised to most reduced) to CDAB prior to H_2O being fed to module C. H_2O conversion would have been high in module C, as the solid in this module was the most reduced of all. However, as the resultant gas mixture enters module D, where the solid is initially more oxidised, part of the H_2 produced in module C is converted back to H_2O, resulting in a lower conversion compared to the previous cycle. By the time the gas stream exits module B, it would have re-established equilibrium with the solids, but since module B was more oxidised than C in cycle 20, the H_2O conversion in cycle 21 would have been lower. For the same reasons, H_2O conversion is expected to be lower in cycles 31, 41 and 61 compared to the majority of the cycles. Since CO is fed to the system after the H_2O half-cycle, the corresponding changes in CO conversion are smaller as the system has partially returned towards the steady-looping state. It can also be seen that the H_2O conversion decrease in cycle 21 is more marked than in cycles 31, 41 and 61. This is due to minor operational oversight, which lead to two consecutive H_2O segments being used for this cycle, as detailed in the experimental record.

In cycle 51 (the start of expt0007), the largest deviation in H_2O conversion was observed. Between expt0006 and expt0007, the sequencing of the modules was unchanged, but the gases were fed to the opposite ends of the overall bed. This effectively created a co-current feeding scenario for 51, which is far less effective than counter-current operation. The H_2O conversion therefore decreased drastically. In fact in co-current operation, the sum of conversions of CO and H_2O should be less than 100% at the operating temperature; whereas in counter-current operation, this can approach 200%.[2] Indeed, the sum of CO conversion from cycle 50 and H_2O conversion from cycle 51 is approximately 85%. In addition, cycle 51 also erroneously had two consecutive H_2O segments, which further reduced the H_2O conversion. Once counter-current operation resumed (from the CO segment of cycle 51 onwards), the conversions of gases quickly recovered to the higher levels expected.

The change in conversion effect is not apparent in cycle 11. This is likely due to the fact that bed conditioning was not complete and steady-cycling had not been established.

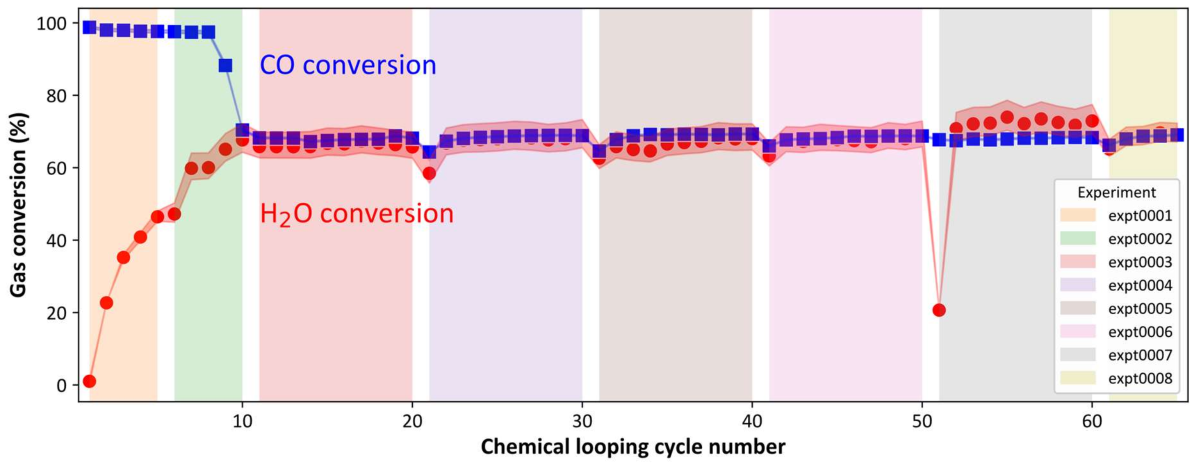

Fig. 10 | LSF gas conversion by MS. Gas conversions for LSF during CL experiments expt0001 to expt0008. Red and blue shaded regions around points represent upper and lower error bounds. Error bounds were calculated from the standard deviations of the signals at the relevant m/z value for each gas of interest during calibration (5% concentration).

Table 5 | Gas flow conditions and conversions for LSF. Summary of gas flow conditions and conversions for LSF CL experiments.

**Experiment**	**0001**	**0002**	**0003**	**0004**	**0005**	**0006**	**0007**	**0008**
**Bed order**	**A**BCD	**A**BCD	D**A**BC	CD**A**B	BCD**A**	**A**BCD	DCB**A**	**A**DCB
**Flow directions**	Water top, CO bottom	Water top, CO bottom	Water top, CO bottom	Water top, CO bottom	Water top, CO bottom	Water top, CO bottom	Water bott, CO top	Water bott, CO top
**Distance from water end (mm)**	34	34	154	274	394	34	394	34
**Number of CL cycles**	5	5	10	10	10	10	10	5
**Average water conversion (%)**	29.2 ± 0.5	60 ± 4	66 ± 4	67 ± 4	66 ± 4	67 ± 4	67 ± 4	68 ± 3
**Average CO conversion (%)**	98.0 ± 0.5	90.2 ± 0.4	68.0 ± 0.2	70.0 ± 0.2	70.4 ± 0.3	68.2 ± 0.2	68.0 ± 0.2	68.1 ± 0.2
**Average oxygen balance (%)**	NA	NA	97 ± 6	96 ± 6	98 ± 6	96 ± 5	89 ± 5	97 ± 4

Rietveld-derived parameters

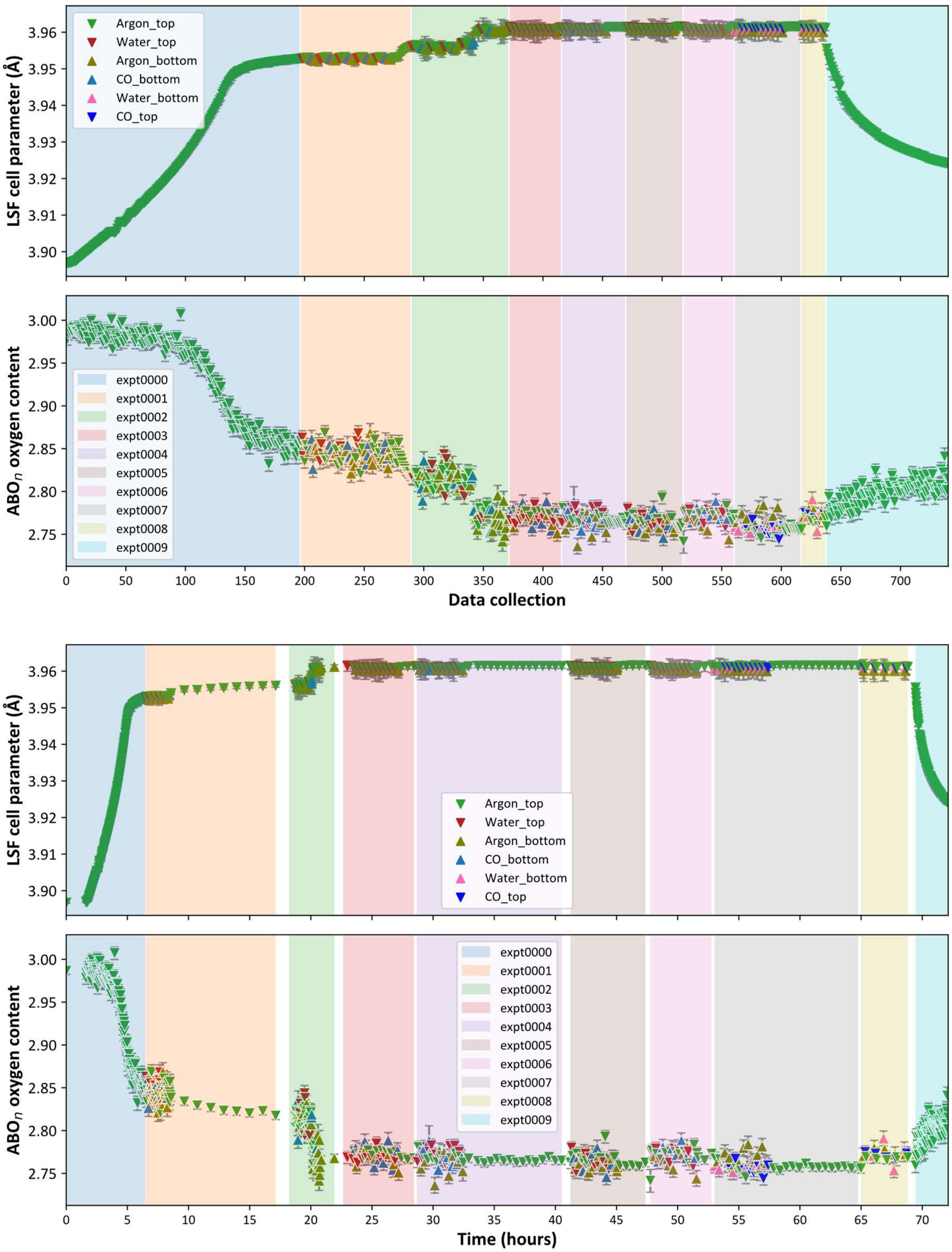

Fig. 11 | Cell parameters and oxygen content of LSF plotted as a function of time. Plots above show the evolution of the cell parameter and oxygen content of LSF as a function of (upper plots) experimental run number and (lower plots) data-collection start time throughout the CL experiments performed. The upper figures are equivalent to Fig. 3a of the main paper, and the lower figures emphasise the longer times spent collecting high-quality data during the long post-CL holds. In this and similar plots later in the SI, points are coloured according to the gas flowing. Oxidising gases (here H_2O) are shown in red shades, reducing (here CO) in blue and the Ar purge in green. A down triangle represents flow to the top of bed module A and an up triangle to the bottom. Error bars plotted are standard uncertainties derived from the Rietveld refinement.

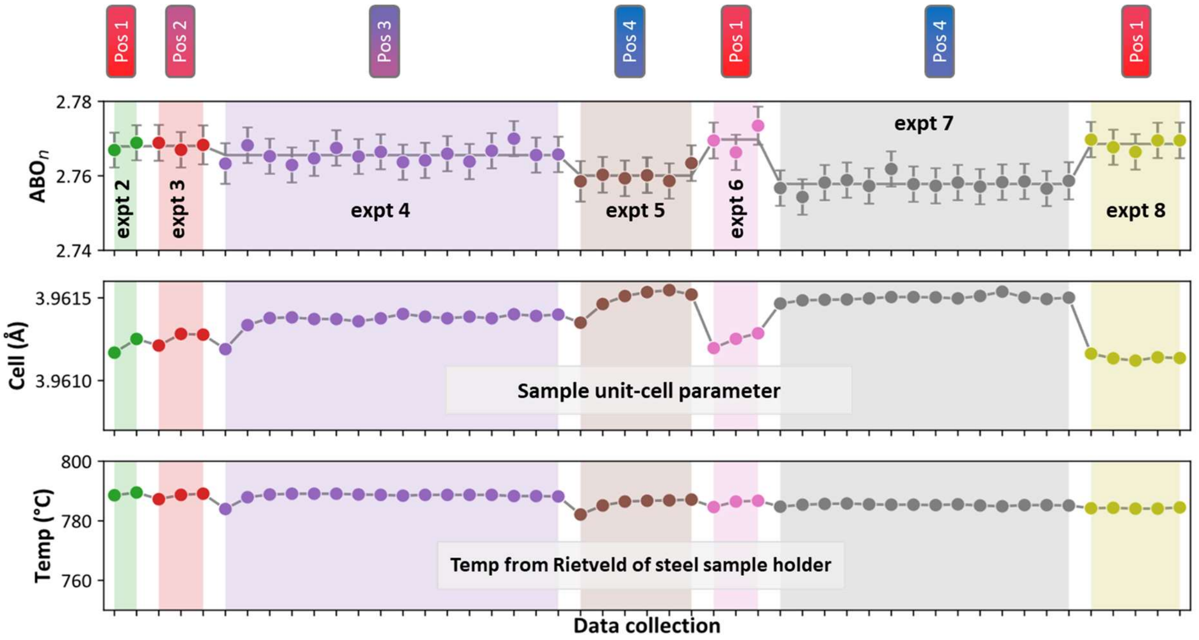

Fig. 12 | Bed parameters under Ar flow long data collections. Plots show the cell parameter and oxygen content of LSF during extended holds under Ar after each CL experiment. Unit cell parameters are corrected from the temperature of the steel sample can as discussed above. Oxygen occupancies remain unchanged during extended holds (average values shown as grey line), indicating no significant transport of oxygen to/from module A and other modules in the bed on these time scales. Unit-cell parameters from expt0004 suggest the bed temperature equilibrates within ~20–30 minutes. Note that the data collection times for each experiment were different and were: expt0002 63 + 38 min; expt0003 31 min; expt0004 30 min; expt0005 30 min; expt0006 2*30+10 min; expt0007 31 min; expt0008 31 min. Standard uncertainties in cell parameters are smaller than the size of the points (typically around 0.00004 Å). Error bars plotted are standard uncertainties derived from the Rietveld refinement.

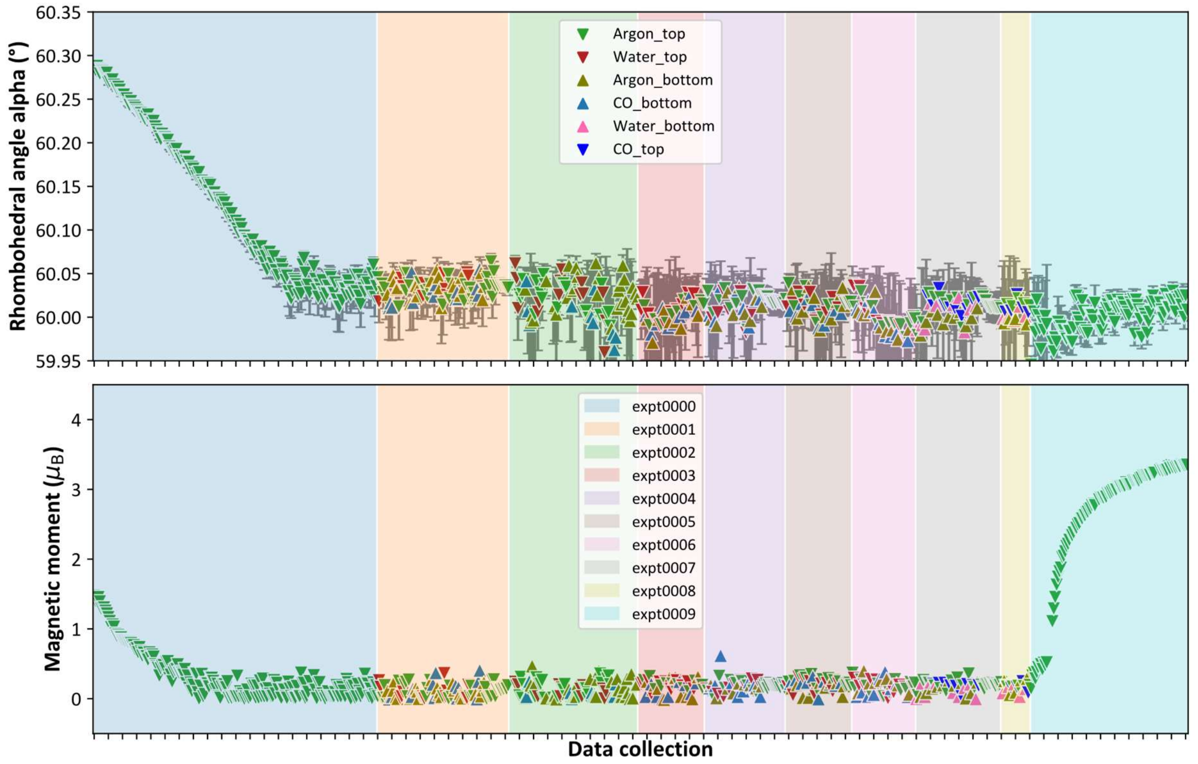

Fig. 13 | Rietveld extracted parameters for LSF. The rhombohedral angle α refines to 60° within uncertainty under CL conditions. In the setting used this corresponds to a metrically cubic cell. Magnetic moments refine to 0.0 μ_B under CL conditions showing LSF remains paramagnetic throughout. The higher magnetic ordering temperature observed on cooling compared to warming is expected due to the increased Fe^{3+} content after CL. Error bars plotted are standard uncertainties derived from the Rietveld refinement.

7. LSFM operando CL experiments

Gas flows and conversion

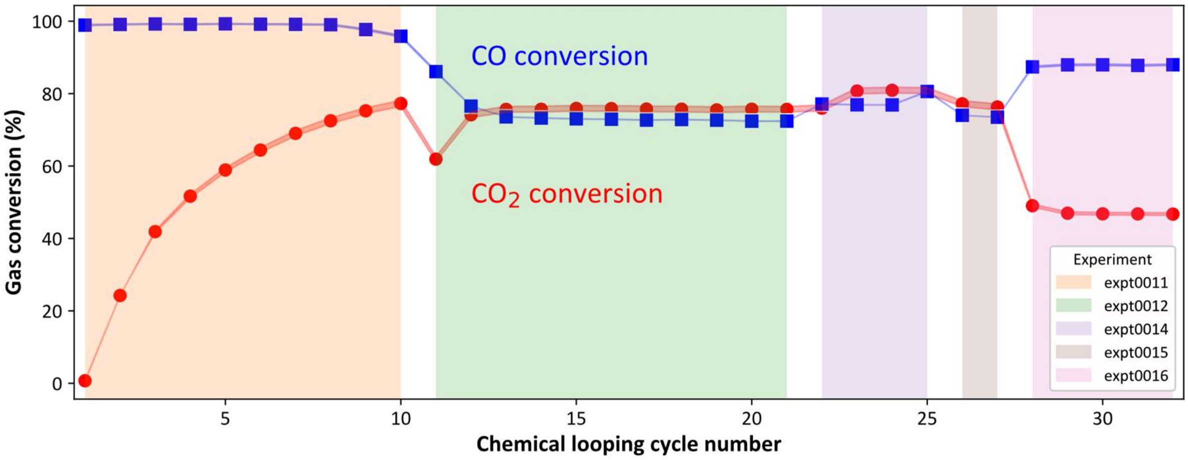

Fig. 14 | LSFM gas conversion by MS. Gas conversions for LSFM during CL experiments 11 to 16. The bed is initially loaded in an oxidised state and has an oxygen content of around 2.97 at the start of expt0011. It therefore shows high CO to CO_2 conversion and low CO_2 to CO. As the overall bed reaches a composition around $ABO_{2.75}$, conversions converge to around 80% for both half reactions in experiments 12 to 15. Experiment 16 used non-balanced conditions to test bed stability with twice the quantity of CO_2 flowed as CO. The overall conversion of CO to CO_2 in the bed-reduction half-cycle is therefore higher than CO_2 to CO in the bed-oxidation half-cycle. Red and blue shaded regions around points represent upper and lower error bounds. Error bounds were calculated from the standard deviations of the signals at the relevant m/z value for each gas of interest during calibration (5% concentration).

Table 6 | Gas flow conditions and conversions for LSFM. Summary of gas flow conditions and conversions for LSFM CL experiments. CO_2 and CO feeds both 5 mol% in argon.

Experiment	0011	0012	0014	0015	0016
Flow directions	CO_2 top, CO bottom	CO_2 top, CO bottom	CO_2 top, CO bottom	CO_2 top, CO bottom	CO_2 top, CO bottom
Flow rate (ml/min, NTP)	400	450	225	225	500
Half cycle duration (min)	2.00	2.16	4.32	4.32	4.00 (CO_2), 2.00 (CO)
Number of cycles	10	11	4	2	5
Average CO_2 conversion (%)	53.6 ± 0.5	74.3 ± 0.8	79.6 ± 0.9	76.7 ± 0.9	47.3 ± 0.4
Average CO conversion (%)	98.7 ± 0.2	74.40 ± 0.01	77.88 ± 0.05	73.75 ± 0.01	87.8 ± 0.2
Average oxygen balance (%)	NA	97 ± 1	98 ± 1	97 ± 1	93 ± 1

Dependence of oxygen content on unit-cell parameters

The relationship between unit-cell parameter and oxygen content shown in Fig. 5 of the main paper was derived by fitting a linear function to oxygen content vs cell parameter for each data collection, with cell parameters corrected for the true sample holder temperature. Despite the scatter, the gradient of 31(3) from expt0014 is within uncertainty of that derived from the conditioning experiment (expt0011) of 32.0(9).

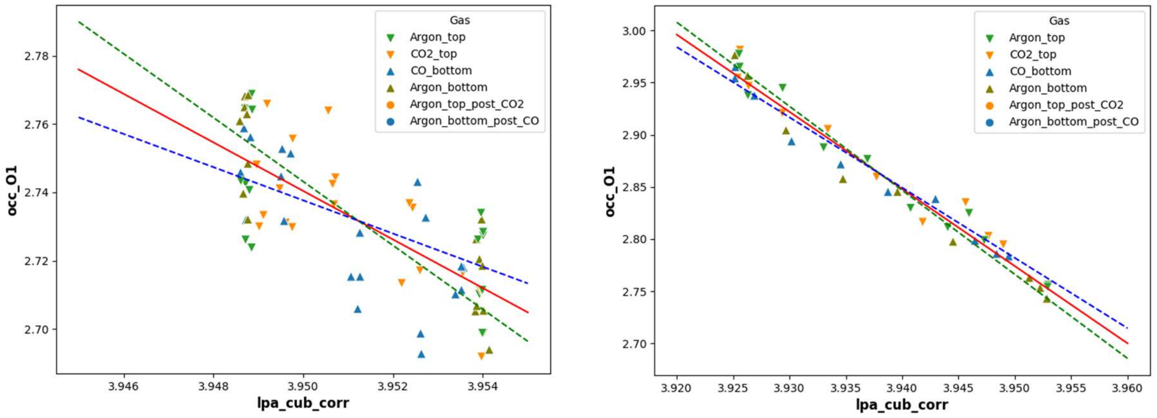

Fig. 15 | Relationship between oxygen content and unit cell parameter in expt0014 (left) and expt0011 (right).

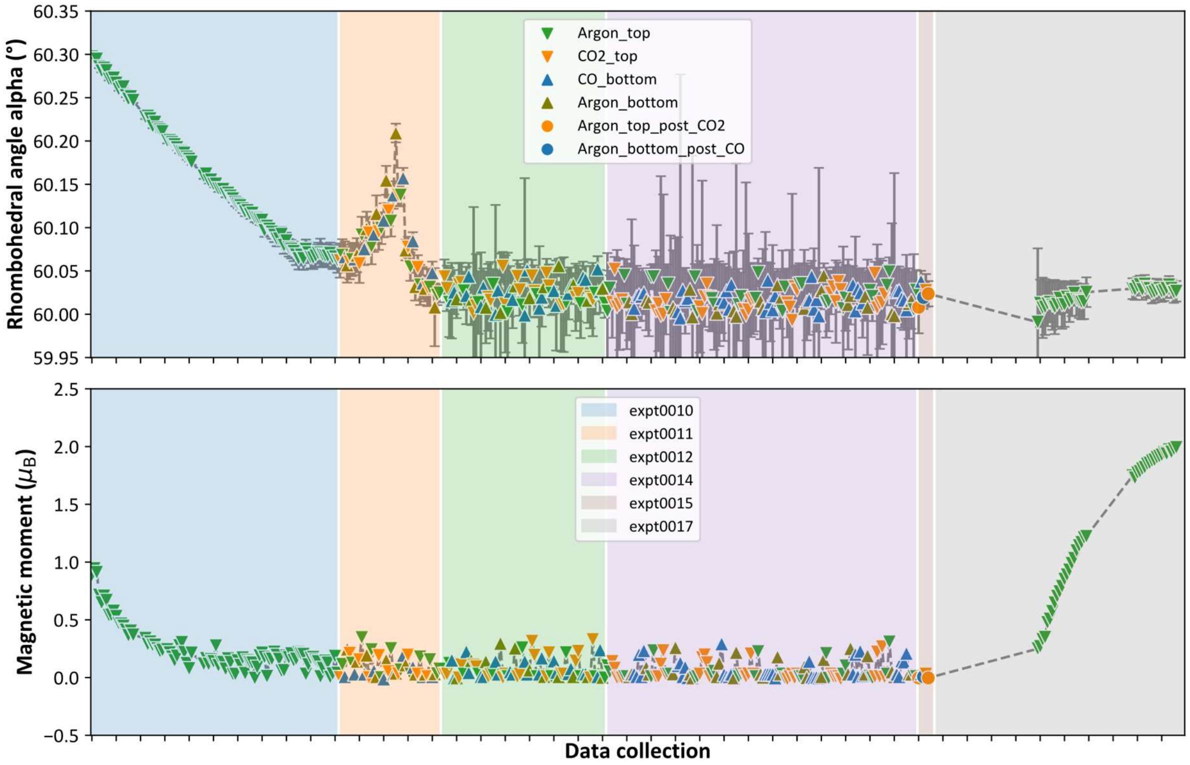

Fig. 16 | Rietveld extracted parameters for LSFM. The rhombohedral angle α refines to 60° within uncertainty under CL conditions implying a cubic average structure. The minor increase in α during expt0011 (conditioning) is due to the neutron beam sampling different regions of the bed with markedly different oxygen contents as the steady-cycling oxygen gradient is established. Magnetic moments fall to 0 μ_B around 275 °C on heating and remain zero within uncertainty under CL showing LSFM remains paramagnetic under operating conditions. Error bars plotted are standard uncertainties derived from the Rietveld refinement.

8. LSFN CL-SMR demonstration experiments

Approximately 0.5 g of LSFN was loaded into a packed bed reactor made of quartz, with a nominal internal diameter of 6 mm. The reactor was connected to a flow system similar to that shown in Fig. 2, which was capable of switching the gas feed directions according to a prescribed control programme. The sample was first oxidised with 1% H_2O balanced in He (100 ml/min at room temperature and pressure; the same flow rate was used throughout this series of experiments) as the reactor was heated in an external electric furnace from room temperature to a set point of 750 °C at a rate of 5 °C/min. The actual sample temperature at the final set point, as measured by a separate K-type thermocouple embedded in the material, was approximately 765 °C. Once the temperature stabilised, a series of counter-current redox CL cycles were carried out as summarised in Table 7.

CL cycles 1–259 were carried out consecutively after the initial oxidation. After cycle 259, operation was halted and the reactor was cooled under helium flow. The reactor was then sealed with parafilm to prevent air ingress and stored under ambient conditions for approximately 10 days. The same bed was subsequently warmed back to 750 °C under flowing helium, then redox

cycles with different reduction and oxidation flow durations performed to investigate the variation of the activity and selectivity of LSFN for the oxidation of CH_4 under different conditions, as shown in Table 7. Before the final set of cycles (381–465), where the flow regime was set to be same as that used in cycles 1–325, the material was oxidised in 1% H_2O for 2 hours isothermally to return the LSFN OCM to a comparable oxidation state as before the commencement of Cycle 1. The external furnace temporarily lost power during Cycle 384, leading to a drop in temperature of the OCM bed to ~500 °C. The experiment was therefore paused until the temperature was restored. This incident did not seem to impact the activity or selectivity of LSFN appreciably.

Table 7 | Gas flow details for LSFN experiments.

Phase	Gases	Gas Flow Direction	Duration (min)				
			Cycles A 1–259	Cycles B 260–325	Cycles C 326–345	Cycles D 346–380	Cycles E 381–465
Pre-reduction inert	helium	↓	1	1	1	1	1
Reduction	5% CH_4 in helium balance	↓	1	1	2	5	1
Post-reduction inert	helium	↓	1	1	1	1	1
Pre-oxidation inert	helium	↑	1	1	1	1	1
Oxidation	1% H_2O in helium balance	↑	5	5	10	15	5
Post-oxidation inert	helium	↑	1	1	1	1	1

The conversion of CH_4 and the selectivity to CO (and therefore syngas) for each redox cycle were calculated as follows:

$$conversion = (amount of CH_4 fed to reactor - amount of unreacted CH_4 leaving the reactor)/(amount of CH_4 fed to reactor)$$

$$selectivity = (amount of CO produced)/(amount of CO produced + amount of CO_2 produced)$$

It is worth noting that the carbon balance of the system was not always close to 1 and in the most extreme cases about 20% of the carbon input was unaccounted for. However, there was no significant carbon deposition observed, either during the subsequent oxidation (see Fig. 17), or on the LSFN material recovered post-experiment. It is likely that the unbalanced carbon accounting was due to a combination of: (1) the duration of the reduction phase deviating slightly from 1 minute due to the control routine operating with a serial protocol for command actions, meaning that the time intervals of valve switching are not precisely controlled (variations of 1–2 seconds have been observed from the leading and trailing edges of the CH_4 signal as measured by the mass spectrometer, which samples data with a rate around 2 Hz); (2) error associated with numerical integration, particularly when the signal to noise ratio is low; (3) slight drift of the mass spectrometer measurement over time; and (4) non-linearity of signal when multiple gas species are present simultaneously.

Conversion and selectivity data are available in SI file LSFN_conversion_selectivity.csv.

The bed materials were recovered after cycle 465 (note that the sample was left in a reduced state at the end of this cycle, as shown in Fig. 17). Post-reaction characterisation by XRD of three different bed section is shown in Fig. 5d of the main text. An SEM image of sample from Pos 3 (most reduced bed section) is shown in Fig. 18. Surface (Ni,Fe) nanoparticles partially embedded in the base perovskite are clearly visible. No carbon whiskers were found in any of the micrographs, further evidencing that no significant carbon deposition took place during the chemical looping methane reforming process.

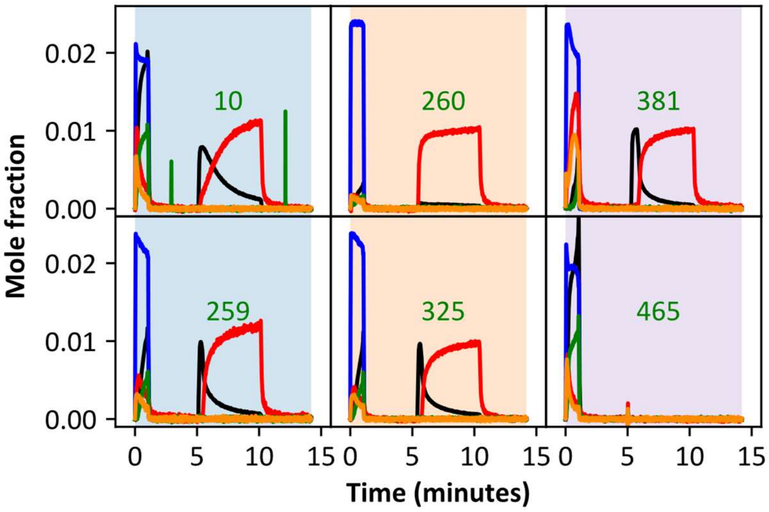

Fig. 17 | LSFN looping. Plot corresponding to Fig. 5c of the main paper, but with gases evolved during the H_2O flow half-cycle included. Red = H_2O, orange = CO_2, green = CO, blue = CH_4, black = H_2. CH_4 is plotted on the right-hand axis which has a numerical scale double that of the left-hand axis. No H_2O half-cycle was performed in the final cycle 465.

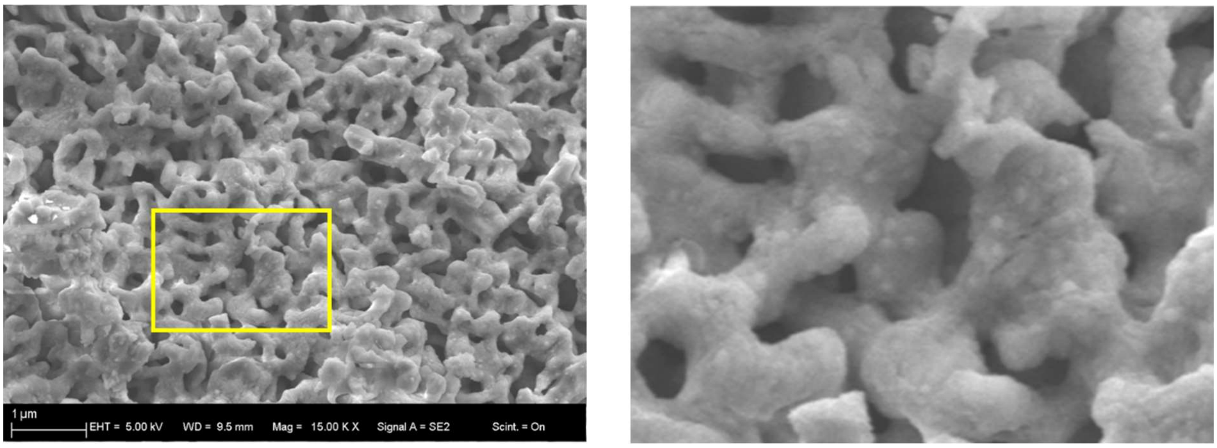

Fig. 18 | LSFN post-looping SEM. SEM micrograph of OCM recovered from the most reduced section of the LSFN bed after cycle 465. (Ni,Fe) particles are visible as paler spots on the surface of the perovskite support in the zoomed region on the right.

9. Supplementary references

1. Pechini MP, inventor Method of Preparing Lead and Alkalne Earth Titanates and Nobates and Coat. US. 1967.
2. Metcalfe IS, Ray B, Dejoie C, Hu W, de Leeuwe C, Dueso C*, et al.* Overcoming chemical equilibrium limitations using a thermodynamically reversible chemical reactor. *Nature Chemistry* 2019, **11**(7)**:** 638-643.
